# Tea-Residue-Derived *Klebsiella pneumoniae* CGMCC 31459: Genomic Insights and Antioxidant Activity of Its Exopolysaccharides

**DOI:** 10.3390/biom15111569

**Published:** 2025-11-07

**Authors:** Yuanyuan Wang, Shengbo Shi, Mingchun Lin, Gangrui Zhang, Longyu Fang, Jinghua Li, Rui Geng, Yuanxue Zheng, Lujiang Hao

**Affiliations:** 1 School of Bioengineering, Qilu University of Technology (Shandong Academy of Sciences), Jinan 250353, China; 10431230832@stu.qlu.edu.cn (Y.W.); 10431230730@stu.qlu.edu.cn (S.S.); 10431240798@stu.qlu.edu.cn (M.L.); 10431221312@stu.qlu.edu.cn (G.Z.); 10431230825@stu.qlu.edu.cn (L.F.); 10431230807@stu.qlu.edu.cn (J.L.); 10431230774@stu.qlu.edu.cn (R.G.); 2 Jinan Qiurong Biotechnology Co., Ltd., Jinan 250353, China; dynwms@126.com

**Keywords:** exopolysaccharides, *Klebsiella pneumoniae*, structure characterization, biological activity, anti-aging activity

## Abstract

Exopolysaccharides (EPSs) are important extracellular metabolites secreted by microorganisms. *Klebsiella pneumoniae* is an opportunistic pathogen widely distributed in the environment, host mucosal and intestinal surfaces, and EPS from *Klebsiella pneumoniae* are of significant interest. Conventional studies have mainly focused on hypervirulent strains, whereas comprehensive investigations of non-hypervirulent strains and their EPS functionalities remain limited. This study employed ST678-type *Klebsiella pneumoniae* CGMCC 31459 as the model to investigate genomic characteristics, EPS structural features, and biological activities. Its genome comprises one chromosome and four plasmids, functionally enriched in carbohydrate metabolism genes, including abundant glycoside hydrolases and glycosyltransferases essential for EPS biosynthesis. Virulence and antimicrobial resistance assessments confirmed the absence of typical hypervirulence loci, indicating genetic stability with low pathogenic and resistance potential. EPS-KP is a weakly acidic, branched heteropolysaccharide composed of glucose, galactose, mannose, and glucuronic acid. EPS-KP exhibited significant antioxidant and anti-aging activities, with a 77.47% (5 mg/mL) superoxide anion scavenging rate and a 30.9% (200 μg/mL) lifespan extension in *Caenorhabditis elegans*, accompanied by enhanced SOD/CAT enzyme activity and reduced lipofuscin accumulation. This integrated genomic and biochemical analysis provides new insights into the safe, non-hypervirulent *Klebsiella pneumoniae* strain and its functional EPS, highlighting its potential for biotechnological applications.

## 1. Introduction

Exopolysaccharides (EPSs) are high-molecular-weight carbohydrate polymers secreted by microorganisms during growth and metabolism. Their structural diversity endows them with functional properties such as adhesiveness, antioxidant activity, and immunomodulatory effects [1,2,3], making them valuable for applications in food additives, drug delivery carriers, cosmetic development, and bioremediation [4,5]. In recent years, the exploration of microbial EPSs with novel structures and superior functional properties has become a research focus in biotechnology. *Klebsiella pneumoniae* is a Gram-negative bacterium widely present in natural environments and human mucosal surfaces, with some strains being pathogenic and capable of causing diseases such as pneumonia and urinary tract infections [6,7]. Recent studies have identified non-pathogenic or low-virulence strains that primarily lose critical virulence factors through genetic deletions or environmental adaptive evolution [8]. Current research predominantly focuses on the pathogenic mechanisms of highly virulent strains, while systematic investigations remain lacking regarding the structural characteristics, secretion patterns, and molecular mechanisms of host interactions associated with EPSs from non-hypervirulent strains [9,10]. The EPS secreted by *Klebsiella pneumoniae* demonstrates unique value in multiple domains, including antioxidation, immunomodulation, antitumor activity, antibacterial effects, and anti-biofilm formation [8,11,12], offering novel perspectives for biomedical applications.

With the rapid development of high-throughput sequencing technologies, genomics has become a core tool for deciphering bacterial metabolic potential, providing crucial support for investigating metabolic pathways, environmental adaptability, and evolutionary mechanisms [13,14]. Through integration of genome annotation, carbohydrate-active enzyme family prediction, and secretion system analysis, the biosynthetic mechanisms of bacterial EPS can be systematically elucidated. Comparative genomics enables the analysis of inter-strain differences by comparing genomic sequences across different bacterial strains, facilitating investigation into the genetic basis of unique phenotypes in specific strains [15]. This approach not only traces evolutionary trajectories but also quantifies environmental adaptation potential through pangenome analysis. The concept of the pan-genome refers to the collection of multiple genomes comprising both core and dispensable components [16]. Pan-genomic analysis can reveal the genetic and evolutionary basis of shared adaptation-related genes within a species or genus [17].

Research methods for polysaccharides primarily focus on analyzing their fundamental structures and chemical compositions. Techniques such as high-performance liquid chromatography (HPLC), Fourier transform infrared spectroscopy (FT-IR), and nuclear magnetic resonance (NMR) enable the determination of monosaccharide composition, glycosidic bond types, and modification characteristics [18,19,20]. Scanning electron microscopy (SEM), atomic force microscopy (AFM), and X-ray crystallography can directly visualize the three-dimensional conformation and surface characteristics of polysaccharides, providing support for investigating structure–activity relationships. For bioactivity evaluation, the application of *Caenorhabditis elegans* (*C. elegans*) models (ROS scavenging, lipofuscin inhibition) enables comprehensive investigation from in vitro chemical activity to in vivo functional validation, offering direct evidence for applications of EPS [19,21]. The multi-level and multi-angle integrated approach provides theoretical support for elucidating the relationship between polysaccharide structure and function.

Recent years have witnessed significant progress in research on *Klebsiella pneumoniae* and its EPS: non-pathogenic *Klebsiella pneumoniae* strains have been successfully engineered through targeted editing of capsular synthesis genes or non-coding RNA-mediated regulation of capsule viscosity, yielding safe variants [10,22]. Their EPS demonstrates remarkable bioactivities, including antioxidant effects, broad-spectrum anti-biofilm properties, and immunomodulatory functions. Novel intervention strategies, including fingolimod (inhibiting *pgaA*/*luxS* gene expression) and engineered phages (targeting the energy metabolism gene *atpA*), have further expanded the application potential of low-virulence strains [23,24]. However, current research still faces significant limitations: ① Structure–function relationship ambiguity: Most studies only indirectly infer the role of EPS active functional groups through chemical modifications, lacking precise control of key parameters such as glycosidic bond configuration and branching degree. ② Unclear dynamic secretion mechanism: The regulatory mechanism of EPS secretion under environmental stress in bacterial strains remains poorly understood, limiting process optimization for industrial production. ③ Insufficient research focus on non-hypervirulent strains: Current studies predominantly concentrate on the virulence mechanisms of highly pathogenic strains, while systematic elucidation of the metabolic networks and EPS functions in non-hypervirulent strains remains lacking [25,26]. Furthermore, the validation of EPS biological activity has been largely confined to in vitro chemical assays, with its regulatory molecular mechanisms still unclear.

In this study, we focused on *K. pneumoniae* CGMCC 31459, an environmental strain with EPS biosynthetic capability that we previously isolated from soaked tea leaf samples. During microbial screening, both cellulase-producing fungi and EPS-producing bacteria were obtained, exhibiting synergistic potential in utilizing cellulose components from tea residues for EPS biosynthesis. Among the bacterial isolates, the strain *K. pneumoniae* CGMCC 31459 showed relatively high EPS yield and stable physicochemical properties and was therefore selected as the representative research subject. The rationale for selecting this strain was to investigate the structural characteristics and biological activities of its EPS, providing fundamental insights into the biosynthesis of bioactive polysaccharides from low-cost plant by-products and establishing a theoretical foundation for the development of environmentally friendly and sustainable polysaccharide production systems. Building upon this foundation, this study focuses on *K. pneumoniae* CGMCC 31459 as the research subject, aiming to elucidate its genomic characteristics and EPS biosynthetic metabolic pathways through whole-genome sequencing and pan-genome analysis. By integrating multidimensional structural characterization (molecular weight, monosaccharide composition, glycosidic linkage configuration) with activity evaluation systems (in vitro free radical scavenging assays and *C. elegans* anti-aging models), we systematically investigate the “structure-function-mechanism” relationship of EPS-KP. This study presents whole-genome and pan-genome analysis of *K. pneumoniae* CGMCC 31459, comprehensively characterizing its polysaccharide structure while evaluating its antioxidant and anti-aging activities. The findings provide novel theoretical foundations for its potential applications in pharmaceutical and industrial fields and offer a scientific basis for developing natural polysaccharide-based anti-aging biologics.

## 2. Materials and Methods

### 2.1. Microbial Information

#### 2.1.1. Strain Information

Experimental strain: The strain used in this study, *Klebsiella pneumoniae* subsp. *pneumoniae* CGMCC 31459 (*K. pneumoniae* CGMCC 31459), which showed relatively high EPS yield and stable physicochemical properties, was isolated from discarded tea residue and is currently preserved at the School of Bioengineering, Qilu University of Technology. The strain has been deposited in the China General Microbiological Culture Collection Center (CGMCC) under the accession number CGMCC 31459. The whole-genome sequencing data has been submitted to the NCBI database with the accession number GCA_049200385.1. The EPS produced by this strain was designated as EPS-KP.

#### 2.1.2. Bacterial and Nematode Culture Conditions

Activation of *K. pneumoniae* CGMCC 31459 was carried out in LB liquid medium (Peptone 10 g/L, Yeast extract 5 g/L, NaCl 10 g/L, pH 7.0) with shaking at 200 rpm at 37 °C. For exopolysaccharide fermentation, the activated *K. pneumoniae* CGMCC 31459 was inoculated into fermentation medium (Maltose 35 g/L, Ammonium sulfate 5 g/L, Sodium chloride 10 g/L, pH 8.0) at an inoculation volume of 10% and cultured under the same shaking conditions at 200 rpm at 37 °C. *Escherichia coli* OP50 (*E. coli* OP50) was also cultured in LB liquid medium with shaking at 200 rpm at 37 °C. After cultivation, the bacterial suspension was spread onto the surface of NGM solid medium and incubated statically at 37 °C for 6–8 h, followed by UV sterilization and storage at 4 °C for later use. *C. elegans* was cultured at 22 °C on NGM plates pre-seeded with UV-sterilized *E. coli* OP50. All reagents used for the culture media were purchased from Sinopharm Chemical Reagent Co., Ltd., Shanghai, China.

### 2.2. Genome Sequencing

#### 2.2.1. Preparation of Sequencing Samples

The cryopreserved strain *K. pneumoniae* CGMCC 31459 was activated in LB medium, and the activated bacterial suspension was subcultured with a 2% (*v*/*v*) inoculation volume. After further cultivation for 24 h under the same conditions, the bacterial cells were harvested by centrifugation at 8000 rpm for 10 min, then washed and resuspended in Phosphate-Buffered Saline (PBS) buffer (Vazyme, Nanjing, China).

Genomic DNA was extracted from bacterial cultures at 2% (*v*/*v*) inoculum using the cetyltrimethylammonium bromide (CTAB) method. Briefly, bacterial cells were harvested by centrifugation and resuspended in CTAB extraction buffer (containing 2% CTAB, 100 mM Tris-HCl pH 8.0, 20 mM EDTA, 1.4 M NaCl, and 0.2% β-mercaptoethanol). The suspension was incubated at 65 °C for 30 min, followed by chloroform:isoamyl alcohol (24:1, *v*/*v*) extraction. The aqueous phase was collected and DNA was precipitated with isopropanol, then washed with 70% ethanol and dissolved in TE buffer. The concentration, purity, and integrity of the extracted DNA were assessed using a Qubit Fluorometer and a NanoDrop Spectrophotometer. High-quality DNA samples were sent to Personal Biotechnology Co., Ltd. (Shanghai, China) for whole-genome sequencing.

#### 2.2.2. Genome Sequencing and Quality Control

The Whole-Genome Shotgun (WGS) strategy was employed, constructing libraries with different insert sizes. Sequencing was performed using both second-generation sequencing (Next-Generation Sequencing, NGS) on the Illumina NovaSeq platform and third-generation single-molecule sequencing on the Oxford Nanopore Technologies (ONT) platform. A total of two libraries were constructed, with detailed library information provided in Table S1. Quality assessment was performed on the raw paired-end FASTQ sequencing data, including statistical analysis of the total read count and GC content. Subsequently, data quality control was conducted using fastp software (v0.23.4), which involved analysis of base content distribution, average read error rate, and nucleotide composition, followed by the removal of adapter sequences and low-quality reads. K-mer analysis was employed to estimate genome size, heterozygosity, and repetitive sequence proportion, thereby obtaining high-quality sequencing data for genome assembly. For third-generation sequencing data, quality metrics, including N50 and read length distribution, were analyzed to ensure data quality.

#### 2.2.3. Genome Assembly

The second-generation sequencing (Illumina) data were processed using A5-miseq (version 20160825) and SPAdes genome assembler (v3.11.1) for K-mer-based error correction and de novo assembly, generating contigs and scaffolds. For third-generation sequencing (Nanopore) data, HGAP4 and CANU (v1.6) were employed to produce scaffold sequences.

The assembled contigs from both sequencing platforms were subsequently integrated to improve assembly continuity. MUMmer (v3) was used for collinearity analysis to validate the assembly results, determine positional relationships between contigs, and fill gaps. Finally, Pilon (v1.22) was applied for assembly polishing to correct remaining errors and generate a complete, high-quality genome sequence.

### 2.3. Genome Analysis

#### 2.3.1. Sequence Typing Identification of *K. pneumoniae* CGMCC 31459

Multilocus sequence typing (MLST), a widely used bacterial typing method for *K. pneumoniae* [27], was employed for strain typing based on multiple housekeeping genes. This method determines the allelic profile and sequence type (ST) by analyzing seven housekeeping genes (*gapA*, *infB*, *mdh*, *pgi*, *phoE*, *rpoB*, and *tonB*). The genomic sequence was submitted to Institut Pasteur MLST databases and software (https://bigsdb.pasteur.fr/klebsiella/, accessed on 18 February 2025) for comparison, which outputs the corresponding ST and allelic information.

#### 2.3.2. Carbohydrate-Active Enzyme Annotation

Carbohydrate-active enzyme (CAZy) analysis was conducted using the hmmscan software (HMMER v3.3.2) [28]. For ORF sequences longer than 80 amino acids, the E-value threshold was set to 1 × 10^−5^, with an alignment length greater than 30% of the database sequence length. For sequences shorter than 80 amino acids, the E-value threshold was set to 1 × 10^−3^, with an alignment length greater than 30% of the database sequence length.

#### 2.3.3. Subcellular Localization Analysis of Protein-Coding Genes

Signal peptide sequences were predicted using SignalP (version 4.1). Transmembrane helix structures were predicted using TMHMM (Server v.2.0). The signal peptide prediction tool SignalP employed neural network and hidden Markov model methods to determine whether protein sequences contained signal peptide structures. Subsequently, the TMHMM tool was used to predict transmembrane structures in protein sequences. Proteins containing signal peptides but lacking transmembrane structures were ultimately identified as secreted proteins.

#### 2.3.4. Functional Annotation of Protein-Coding Genes

Protein-coding gene sequences were aligned against the eggNOG (COG) database using DIAMOND BLASTP (v2.0.15), with an alignment threshold set at 1 × 10^−6^. The best hit from eggNOG was assigned to the corresponding protein-coding gene. KEGG Orthology (KO) annotation was performed using the KAAS (KEGG Automatic Annotation Server, Version 2.1) system, employing the “For Prokaryotes” gene set and the bi-directional best hit (BBH) method. The annotated KOs were then mapped to KEGG Pathways. Gene Ontology (GO) annotation was conducted using BLAST2GO with default parameters, and GOSlim annotation was generated using map2slim (OWLTools v0.3.5).

#### 2.3.5. Genome Circular Map Construction

The genome sequence, gene predictions, and non-coding RNA predictions were compiled into a standard GenBank format file. The cgview tool was then used to generate the genome circular map.

#### 2.3.6. Analysis of Virulence, Antimicrobial Resistance, and Mobile Genetic Elements

To comprehensively characterize the genomic features of *K. pneumoniae* CGMCC 31459, virulence factors, antimicrobial resistance (AMR) genes, and mobile genetic elements were systematically analyzed. Virulence genes were identified using the Virulence Factors Database (VFDB, https://www.mgc.ac.cn/cgi-bin/VFs/v5/main.cgi, accessed on 16 October 2025), where the assembled genome was compared against the VFDB core dataset to annotate and classify putative virulence determinants [29]. AMR gene annotation was performed using both the Comprehensive Antibiotic Resistance Database (CARD, https://card.mcmaster.ca/analyze/rgi, accessed on 15 October 2025) and ResFinder (https://cge.food.dtu.dk/services/ResFinder/, accessed on 15 October 2025) [30,31]. The predicted coding sequences were first aligned to the CARD database to identify potential resistance genotypes and then validated against ResFinder; only high-confidence AMR genes identified by both databases were retained for further analysis. Mobile genetic elements, including prophages, genomic islands, and plasmids, were annotated using PHASTER (https://phaster.ca/, accessed on 16 October 2025) [32,33], IslandViewer4 (https://www.pathogenomics.sfu.ca/islandviewer/, 16 October 2025) [34], respectively. Genomic islands were predicted through the integration of four algorithms (IslandPath-DIMOB, SIGI-HMM, IslandPick, and Islander), and only regions supported by at least two methods and larger than 10 kb were considered high-confidence islands for subsequent analysis.

### 2.4. Pan-Genome Analysis

#### 2.4.1. *Klebsiella pneumoniae* Genome Collection

The study included the strain *K. pneumoniae* CGMCC 31459 (sequenced in this work) along with 12 additional *K. pneumoniae* strains with reported high virulence or multidrug resistance. The genomic data of these 12 strains were retrieved and downloaded from the NCBI database. The strain and GCA accession number used in this study are as follows:

*Klebsiella pneumoniae* subsp. *pneumoniae* NTUH-K2044 (GCA_000009885.1), *Klebsiella pneumoniae* subsp. *pneumoniae* MGH 78578 (GCA_000016305.1), *Klebsiella pneumoniae* subsp. *pneumoniae* HS11286 (GCA_000240185.2), *Klebsiella pneumoniae* UCI 56 (GCA_000694815.1), *Klebsiella pneumoniae* subsp. *pneumoniae* ATCC 43816 (GCA_000742755.1), *Klebsiella pneumoniae* XH209 (GCA_001699105.2), *Klebsiella pneumoniae* GN-2 (GCA_001939885.2), *Klebsiella pneumoniae* QS17-0029 (GCA_003073235.1), *Klebsiella pneumoniae* subsp. *Pneumoniae* CR-HvKP1 (GCA_005853785.1), *Klebsiella pneumoniae* K28074 (GCA_025397975.1), *Klebsiella pneumoniae* K30821 (GCA_025725705.1), *Klebsiella pneumoniae* subsp. *pneumoniae* HVKP1 (GCA_030020765.1).

#### 2.4.2. Pan-Genome and Comparative Genomic Analysis

Pan-genome analysis was conducted using the IPGA online platform (https://nmdc.cn/ipga/, accesed on 23 December 2024) [35]. Prior to analysis, genome sequences underwent quality control with the following criteria: only sequences with >90% completeness and <5% contamination were retained for downstream analyses. Average nucleotide identity (ANI) and synteny analyses were performed, with strains exhibiting ANI values >95% being classified as the same species. The pan-genome of 13 *K. pneumoniae* strains was analyzed using IPGA with multiple analytical modules, including PANOCT, OrthoMCL, Roary, panX, OrthoFinder, Panaroo, and PPanGGoLiN. The analysis parameters were set as follows: Identity = 70, Ratio (core) = 0.95, and Support = −1.

#### 2.4.3. KEGG Pathway Analysis of *K. pneumoniae* CGMCC 31459

Predicted proteins from the *K. pneumoniae* CGMCC 31459 whole genome were obtained through BlastKOALA for KEGG Orthology (KO) functional annotation. The metabolic and regulatory pathways of *K. pneumoniae* CGMCC 31459 were then visualized using iPath v3 based on KO identifiers [13,36].

### 2.5. EPS Secretion Study of K. pneumoniae CGMCC 31459

*K. pneumoniae* CGMCC 31459 was inoculated into LB liquid medium and incubated at 37 °C with shaking at 200 rpm for 8 h. The culture was then transferred to fermentation medium and further incubated at 37 °C with shaking at 200 rpm. The growth curve of *K. pneumoniae* CGMCC 31459 was monitored, with fermentation broth samples collected every four hours to analyze EPS content and quantify temporal variations in EPS production. The EPS yield in the fermentation broth of *K. pneumoniae* CGMCC 31459 at different fermentation times was determined using the phenol-sulfuric acid method. Samples were taken at three time points: the early logarithmic phase, the stationary phase, and the time of maximum EPS production. The cultures were centrifuged at 4 °C at 5000 rpm for 15 min. The supernatants were discarded, and the bacteria were resuspended in 10 mM phosphate-buffered saline (PBS, pH 7.4), followed by two additional centrifugation and washing steps under the same conditions to obtain the bacterial precipitate.

Bacterial samples were fixed with 4% paraformaldehyde at 25 °C for 30 min and centrifuged at 4 °C at 2000 rpm for 10 min to collect the precipitate. After washing twice with PBS to remove residual fixative, 500 µL of FITC-ConA solution (20 µg/mL) was added, and the mixture was incubated in the dark for 30 min. The precipitate was then centrifuged, washed twice with PBS, and stained with 500 µL of PI solution (50 µg/mL) under the same conditions. Following an additional wash to remove excess dye, the bacterial precipitate was resuspended in 200 µL PBS, and 15 µL of the suspension was mounted on a glass slide with a coverslip for fluorescence microscopy analysis [37].

### 2.6. Structural Characterization of EPS-KP

#### 2.6.1. Extraction, Isolation, and Purification of EPS-KP

Cryopreserved *K. pneumoniae* CGMCC 31459 was activated and inoculated into LB liquid medium. After cultivation, the culture was transferred into the fermentation medium and incubated under constant temperature with shaking for 36 h. Bacterial cells were removed by centrifugation at 5000 rpm for 20 min, and the supernatant was concentrated to one-third of its original volume using a rotary evaporator. With continuous stirring on a magnetic stirrer (Shanghai Jingke, Shanghai, China), three volumes of ethanol were slowly added to the concentrated supernatant, and the mixture was kept at 4 °C overnight to ensure complete precipitation. The precipitate was collected by centrifugation at 5000 rpm for 20 min. Sevage reagent (chloroform:n-butanol = 4:1, *v*/*v*) was prepared, and the crude EPS was treated using the Sevage method to remove organic solvents and denatured proteins. The EPS solution was analyzed by UV-Vis spectrophotometry at 260 nm and 280 nm to confirm the absence of absorption peaks, indicating complete protein removal. Further purification was performed using DEAE-52 cellulose ion-exchange chromatography and Sephadex gel filtration chromatography. The purified EPS was dialyzed against deionized water using a semipermeable membrane (8000–14,400 Da). Finally, the EPS was freeze-dried under vacuum to obtain the purified EPS product, which was stored at 4 °C.

#### 2.6.2. Molecular Weight Determination of EPS-KP

An appropriate amount of EPS-KP was dissolved in 0.1 M NaNO_3_ aqueous solution (containing 0.02% NaN_3_) to a final concentration of 1 m *C. elegans*, followed by filtration through a 0.45 μm membrane prior to analysis. The molecular weight was determined using a gel permeation chromatography–refractive–index-multi-angle laser light scattering (GPC-RI-MALLS) system (U3000, Thermo, Waltham, MA, USA) equipped with tandem gel-filtration columns (Ohpak SB-805 HQ and SB-803 HQ, 300 × 8 mm each). The analysis was performed at 45 °C with an injection volume of 100 μL, using mobile phase A (0.02% NaN_3_ in 0.1 M NaNO_3_) at a flow rate of 0.6 mL/min (isocratic elution) [38].

#### 2.6.3. Monosaccharide Composition Analysis of EPS-KP

The EPS-KP sample was placed in a chromatography vial and hydrolyzed with 1 mL of 2 M trifluoroacetic acid at 121 °C for 2 h. After repeated washing with methanol and reconstitution in sterile water, the sample was filtered through a 0.22 μm membrane. Monosaccharide composition was analyzed using a Thermo ICS 5000+ ion chromatography system with a Dionex™ Carbopac™ PA20 column (Thermo, Waltham, MA, USA). The mobile phases consisted of (A) H_2_O, (B) 0.1 M NaOH, and (C) 0.1 M NaOH + 0.2 M NaOAc, delivered at 0.5 mL/min. The column temperature was maintained at 30 °C [18].

#### 2.6.4. Fourier Transform Infrared Spectroscopy (FTIR) Analysis of EPS-KP

A 1 mg aliquot of purified EPS-KP was thoroughly mixed with 100 mg of desiccated KBr powder in an agate mortar and pressed into a 1 mm-thick pellet using a hydraulic press (Tianjin Tianguang, Tianjin, China). FTIR spectra were acquired on a Nicolet iZ-10 (Thermo Fisher Scientific, Waltham, MA, USA) spectrometer, scanning the wavelength range of 4000–450 cm^−1^ to characterize functional groups [20].

#### 2.6.5. Methylation Analysis of EPS-KP

3 mg of EPS-KP was first dissolved in 500 μL of DMSO. After adding 1 mg of NaOH, the mixture was incubated for 30 min, followed by reaction with 50 μL of methyl iodide solution for 1 h. The solution was then extracted with 1 mL of water and 2 mL of dichloromethane. The mixture was vortexed and centrifuged to remove the aqueous phase, with this extraction process repeated three times. The lower dichloromethane phase was collected and dried under nitrogen gas. Subsequently, 100 μL of 2M trifluoroacetic acid was added, and the sample was reacted at 121 °C for 90 min, then evaporated to dryness at 30 °C. The sample was then sequentially treated with 50 μL of 2M ammonia water and 50 μL of 1M NaBD_4_, reacting at room temperature for 2.5 h before quenching with 20 μL of acetic acid. After drying under nitrogen, the sample was washed twice with 250 μL of methanol. Finally, 250 μL of acetic anhydride was added, and the reaction proceeded at 100 °C for 2.5 h. The reaction was terminated by adding 1 mL of water and letting it stand for 10 min, followed by extraction with 500 μL of dichloromethane. The aqueous washing step was repeated three times, and the lower organic phase was collected for GC-MS analysis. The analysis was performed using an Agilent 6890A-5977B GC-MS system (Agilent Technologies Inc., Santa Clara, CA, USA) with the following parameters: quadrupole temperature at 110 °C, ionization energy at 50 eV, transfer line temperature at 210 °C, and mass scanning range of *m*/*z* 50–350 [39]. The methylation analysis was performed according to the procedure for neutral polysaccharides, without a carboxyl reduction step; therefore, the obtained GC–MS data mainly reflect the linkage patterns of neutral sugar residues.

#### 2.6.6. Scanning Electron Microscopy of EPS-KP

For SEM observation, the EPS-KP sample was first passed through a 100-mesh sieve. A small amount of the sieved sample was placed on conductive carbon tape and sputter-coated with gold. The sample was then examined using a Zeiss Merlin Compact high-resolution field emission scanning electron microscope (Carl Zeiss Microscopy GmbH, Jena, Germany). Imaging was performed with an acceleration voltage ranging from 0.02 to 30 kV and at magnifications between 1000× and 10,000×.

### 2.7. EPS-KP In Vitro Antioxidant Activity

#### 2.7.1. Hydroxyl Radical (-OH) Scavenging Assay

The hydroxyl radical scavenging capacity was determined using the Fenton reaction by measuring the OD value of the product formed from the reaction between salicylic acid and -OH [40]. The reaction system was configured by mixing 0.1 mL of FeSO_4_ solution (9 mmol/L), 0.1 mL of salicylic acid-ethanol solution (9 mmol/L), and 0.1 mL of EPS-KP test sample solution at different concentrations. Then, 0.1 mL of H_2_O_2_ solution (1 mmol/L) was added to initiate the reaction. The reaction system was maintained in a water bath at 37 °C for 30 min, and the absorbance was measured at 510 nm and recorded as AX.

(1) Hydroxyl radical scavenging rate (%) = [1 − (AX − A1)/A0] × 100

#### 2.7.2. ABTS Radical Scavenging Assay

To 20 μL of EPS test samples at different concentrations, 200 µL of ABTS solution (absorbance value of 0.70 ± 0.02 at 734 nm) was added and mixed. The reaction was performed at 25 °C for 6 min [41]. The absorbance was measured at 734 nm and recorded as AX.

(2) ABTS free radical scavenging rate (%) = [1 − (AX − A1)/A0] × 100

#### 2.7.3. DPPH Radical Scavenging Assay

To 50 μL of EPS test samples at different concentrations, 25 μL of DPPH-ethanol solution (0.4 mmol/L) and 100 μL of deionized water were added sequentially. The reaction system was mixed well and then incubated in the dark at 30 °C for 30 min [42]. The absorbance was measured at 517 nm and recorded as AX.

(3) DPPH free radical scavenging rate (%) = [1 − (AX − A1)/A0] × 100

#### 2.7.4. Superoxide Anion (O_2_^−^) Radical Scavenging Assay

The O_2_^−^ scavenging capacity of EPS was determined using the o-triol autoxidation assay. For this, 3.2 mL of deionized water was added to 4.5 mL of Tris-HCl buffer (50 mmol/L, pH 8.2), and 1.0 mL of EPS test samples at different concentrations were tested at 25 °C for 4 min [43]. The absorbance was measured at 325 nm and recorded as AX.

(4) Superoxide Anion scavenging rate (%) = [1 − (AX − A1)/A0] × 100

The antioxidant activity of EPS-KP was evaluated at final concentrations of 0.4, 0.8, 1.2, 1.6, 2.0, 3.0, and 5.0 mg/mL, where AX denotes the absorbance of the EPS reaction solutions at different concentrations, A0 denotes the absorbance measured using deionized water, and A1 denotes the background absorbance of the EPS solution at the corresponding concentration. All experiments were repeated thrice. After obtaining free radical scavenging rate, data were processed using GraphPad Prism 10.2.3, and nonlinear regression was performed based on the log(inhibitor) vs. normalized-response–variable-slope model to determine the dose–response relationship.

### 2.8. C. elegans Anti-Aging Experiments

#### 2.8.1. Nematodes Synchronization

The N2 wild-type *C. elegans* and *E. coli* OP50 used in this study were obtained from SunyBiotech (SunyBiotech, Fujian, China). The nematodes were cultured at a constant temperature of 22 °C on nematode growth medium (NGM) plates seeded with *E. coli* OP50. After cultivation, the nematodes were washed with M9 buffer to remove residual bacteria and treated with a nematode lysis solution (NaOH: NaClO: H_2_O = 1:5:10) to obtain synchronized eggs. The eggs were resuspended in M9 buffer and transferred to fresh NGM plates pre-seeded with *E. coli* OP50. After 48 h of incubation at 22 °C, L4-stage nematodes were obtained for subsequent experiments [44,45].

#### 2.8.2. Lifespan Assay

Synchronized L4-stage *C. elegans* were transferred to NGM plates pre-seeded with *E. coli* OP50 and supplemented with different concentrations of EPS-KP (100 µg/mL, 200 µg/mL, 300 µg/mL) or without EPS-KP (control, in which water was used instead of the EPS-KP). For each group, 30 nematodes were randomly selected, and three biological replicates were performed. Starting from day 0, nematode mortality was recorded daily. To prevent newly hatched progeny from interfering with the data, surviving nematodes were transferred to fresh NGM plates every 48 h. Nematodes were considered dead if they showed no response after three consecutive touches with a platinum wire pick.

Survival data were analyzed using the Kaplan–Meier method, and statistical significance between groups was evaluated with the log-rank (Mantel–Cox) test. Mean lifespan and percentage change data were analyzed using one-way ANOVA followed by Tukey’s multiple comparison test. All statistical analyses and graphing were performed using GraphPad Prism 10.2.3. The same statistical methods were applied to the heat stress and oxidative stress assays described below.

#### 2.8.3. Heat Stress and Oxidative Stress Tests

L4-stage *C. elegans* were cultured for two days on NGM plates containing different EPS concentrations or control plates before stress assays. For the heat stress test, nematodes were exposed to 37 °C, and mortality was recorded hourly until all nematodes died. For the oxidative stress test, nematodes were placed on NGM plates containing 5 mM H_2_O_2_, and mortality was recorded every 30 min until all nematodes died. Each experiment was performed in triplicate with 30 randomly selected L4-stage nematodes per treatment [19].

#### 2.8.4. Lipofuscin Level Measurement

L4-stage *C. elegans* were cultured for five days on NGM plates with or without EPS. They were then washed with M9 buffer to remove residual bacteria and anesthetized using levamisole hydrochloride. Lipofuscin fluorescence was visualized using a confocal laser microscope, and fluorescence intensity was quantified using ImageJ software (v1.52). The experiment was repeated three times, with 30 nematodes per treatment [21]. The data for lipofuscin accumulation, reactive oxygen species (ROS) levels, and antioxidant enzyme activities (CAT and SOD) were analyzed using one-way ANOVA followed by Tukey’s multiple comparison test. All statistical analyses and graphing were performed using GraphPad Prism 10.2.3, and results were expressed as mean ± standard deviation (SD).

#### 2.8.5. ROS Content Measurement

L4-stage *C. elegans* were cultured for five days on NGM plates with or without EPS, washed with M9 buffer, and treated using the Beyotime Reactive Oxygen Species Assay Kit (Beyotime Biotechnology, Shanghai, China). The nematode suspension was incubated with the DCFH-DA probe for 3 h at 22 °C, washed with M9 buffer to remove unbound probe, and anesthetized with levamisole hydrochloride. ROS levels were assessed by measuring fluorescence intensity using confocal microscopy (Leica SP8, Leica Microsystems, Wetzlar, Germany) and ImageJ software. Each treatment included three replicates of 30 nematodes.

#### 2.8.6. SOD, CAT Activity Assays

L4-stage *C. elegans* were cultured for five days on NGM plates with or without EPS, washed with M9 buffer, and homogenized in PBS. Superoxide dismutase (SOD) and catalase (CAT) activities were measured using the Beyotime Total SOD Assay Kit with NBT and the Beyotime CAT Assay Kit, respectively (Beyotime Biotechnology, Shanghai, China). All experiments were repeated five times.

## 3. Results

### 3.1. Genome Sequencing and Analysis of K. pneumoniae CGMCC 31459

#### 3.1.1. Functional Annotation Analysis

Genomic annotation of *K. pneumoniae* CGMCC 31459 revealed that its genome consists of one chromosome and four plasmids, containing a total of 5079 predicted open reading frames (ORFs). The chromosome contains 4848 ORFs with a total coding sequence length of 4,486,524 bp, representing a coding density of 86.36%. The chromosomal region demonstrates high gene density (0.933 genes/kb) and GC content (58.99%), with an average ORF length of 925.44 bp. The plasmids exhibited marked heterogeneity in genomic characteristics. Plasmid1 contained 221 ORFs with a coding region coverage of 82.75%, while Plasmid2-4 demonstrated progressively diminishing coding capacity. Notably, Plasmid4 carried only 2 ORFs with a substantially reduced coding coverage of 29.13%. The gene density across these plasmids ranged from 0.798 to 1.107 genes/kb, with GC content varying significantly from 38.07% to 53.80% (Table S2). Non-coding RNA analysis identified 9 copies of 5S rRNA, 8 copies each of 16S rRNA and 23S rRNA, along with 86 tRNA genes. Collectively, these RNA elements account for 0.83% of the total genome length (Table S3).

#### 3.1.2. Typing and Identification of *K. pneumoniae* CGMCC 31459

Multilocus sequence typing (MLST) analysis of *Klebsiella pneumoniae* CGMCC 31459 was performed by examining sequence variations in seven housekeeping genes (*gapA*, *infB*, *mdh*, *pgi*, *phoE*, *rpoB*, and *tonB*) (Table S4). The strain was classified as sequence type ST678, exhibiting the following allele profile: *gapA*-2 (450 bp), *infB*-3 (318 bp), *mdh*-1 (477 bp), *pgi*-1 (432 bp), *phoE*-109 (420 bp), *rpoB*-56 (501 bp), and *tonB*-18 (414 bp). These molecular typing results provide crucial insights into the strain’s genetic background and establish fundamental data for subsequent epidemiological investigations and evolutionary analyses.

#### 3.1.3. Carbohydrate-Active Enzyme Analysis

Genomic analysis using the Carbohydrate-Active Enzymes (CAZy) database revealed remarkable diversity in carbohydrate-metabolizing enzymes encoded by *K. pneumoniae* CGMCC 31459, with glycoside hydrolases (GHs, 69 members) and glycosyl transferases (GTs, 33 members) representing the predominant families. GH enzymes primarily facilitate glycosidic bond hydrolysis and substrate breakdown, generating essential monosaccharide precursors and metabolic energy while enabling carbon acquisition through degradation of plant- or host-derived polysaccharides; these enzymes may additionally participate in extracellular polysaccharide remodeling to modulate biological activity. In contrast, GT enzymes principally mediate extracellular polysaccharide synthesis and modification, serving as core components of biosynthetic pathways for complex carbohydrate structures.

**Figure 1:**
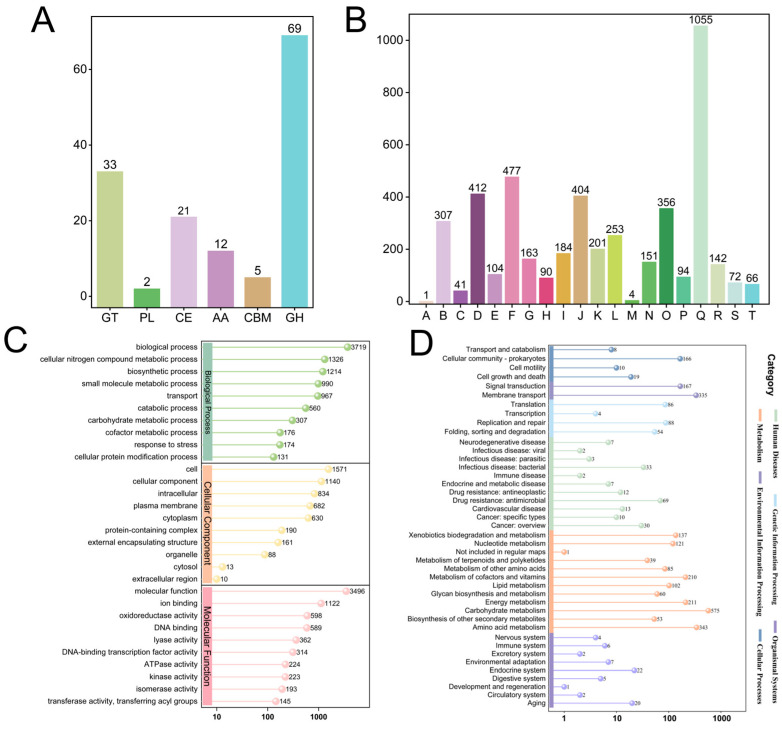
Genomic feature analysis of *K. pneumoniae* CGMCC 31459; (**A**) CAZy functional classification chart for Chr; (**B**) Bacterial eggNOG (COG) functional classification chart—for more details, see the Supplementary Materials; (**C**) GO Slim annotation chart; (**D**) KEGG pathway statistics chart.

The genomic profile also included carbohydrate esterases (CEs, 21 members), auxiliary activities (AAs, 12 members), carbohydrate-binding modules (CBMs, 5 members), and polysaccharide lyases (PLs, 2 members) (Figure 1A). CE family enzymes primarily modify carbohydrate compounds through deacetylation and ester bond hydrolysis, thereby regulating polysaccharides’ physicochemical properties, while their interaction with AA family enzymes potentially contributes to oxidative stress response and antioxidant activity—a feature closely associated with extracellular polysaccharides’ (EPS) antioxidative characteristics. PLs and CBMs participate in polysaccharide degradation and specifically recognize/bind particular carbohydrate domains, providing auxiliary functions for substrate enzyme activity. The synergistic actions of these enzyme systems collectively establish the molecular foundation for EPS synthesis and biological functionality, suggesting EPS-KP possesses significant potential value.

#### 3.1.4. Functional Annotation of Protein-Coding Genes

We performed systematic functional annotation of protein-coding genes in the bacterial genome. Classification using eggNOG (COG) revealed that 94.41% of genes were categorized into 20 functional groups (Figure 1B), predominantly including carbohydrate transport and metabolism (9.84%), amino acid transport and metabolism (8.50%), and energy production (6.33%), demonstrating its active metabolic capacity and environmental adaptation potential. Notably, 21.76% of genes were classified as “function unknown”, suggesting possible species-specific functional modules or novel genes. GO enrichment analysis revealed significant enrichment of this strain in carbohydrate metabolic processes and biosynthetic processes, particularly demonstrating high expression of transferase activity and oxidoreductase activity, suggesting its robust capacity for EPS synthesis and modification. Concurrently, the enrichment of genes associated with external encapsulating structures directly supports EPS biosynthesis functionality, while the active response to stress and transport systems provide both environmental adaptability and precursor supply assurance for EPS production (Figure 1C). KEGG pathway enrichment analysis demonstrated significant characteristics in carbohydrate metabolism networks, with particular enrichment in glycan biosynthesis and metabolism pathways that are closely associated with EPS production capability. Concurrently, the active energy metabolism and metabolism of cofactors and vitamins pathways provide essential ATP and sugar nucleotide precursors for EPS biosynthesis. Multiple genes encoding sugar transporters detected in membrane transport pathways likely participate in EPS precursor uptake and translocation, while the enriched signal transduction pathways may regulate EPS synthesis and secretion. These collective features establish the molecular foundation for functional EPS production in this strain (Figure 1D). Comprehensive analysis revealed that the genomic basis of metabolic diversity and EPS biosynthesis mechanisms collectively constitute the molecular framework for EPS synthesis.

#### 3.1.5. Subcellular Localization Analysis of Protein-Coding Genes

Genomic subcellular localization analysis revealed significant functional divergence in secretory system components (Table S5). The chromosome encodes 386 signal peptide-containing proteins (7.96% of total encoded proteins) and 1160 transmembrane proteins (23.93%), including 305 classical secretory proteins (6.29%), indicating its dominant role in secretory pathway functions. The plasmids exhibited distinct functional specialization: Plasmid 1 contains 9 signal peptide proteins (4.07%) and 4 transmembrane proteins (18.55%), while Plasmids 2 and 4 each possess a single transmembrane protein (accounting for 25% and 50% of their respective protein-coding capacity) without detectable signal peptides, and Plasmid 3 completely lacks these features. This functional partitioning among genomic elements reflects evolutionary adaptations to different selective pressures, providing crucial insights into deciphering the strain’s specialized secretory systems and membrane biology characteristics.

#### 3.1.6. Genome Circle Plot Construction

**Figure 2:**
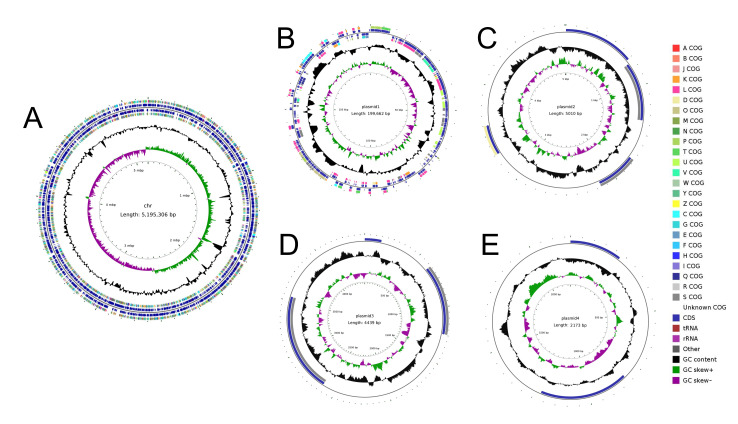
Circular genome map of *K. pneumoniae* CGMCC 31459: (**A**) chromosome; (**B**) plasmid 1; (**C**) plasmid 2; (**D**) plasmid 3; (**E**) plasmid 4. From innermost to outermost: the first ring represents the scale; the second ring represents GC skew; the third ring shows GC content; the fourth and seventh rings represent the COG classifications of each CDS; the fifth and sixth rings indicate the locations of CDSs, tRNAs, and rRNAs on the genome.

The complete genomic circle plot of *K. pneumoniae* CGMCC 31459, integrating its genetic architecture, is presented in Figure 2. The genome consists of one circular chromosome and four plasmids, with sizes of 5,195,306 bp (Figure 2A), 199,662 bp (Figure 2B), 5010 bp (Figure 2C), 4439 bp (Figure 2D), and 2173 bp (Figure 2E), respectively.

#### 3.1.7. Virulence, Antimicrobial Resistance, and Mobile Genetic Elements Analysis

Based on the VFDB virulence factor prediction, a total of 93 virulence-associated genes were identified in *Klebsiella pneumoniae* CGMCC 31459, which were distributed across six major functional categories, including adherence, antiphagocytosis, iron uptake, and secretion systems (Table S6). However, several hallmark determinants characteristic of hypervirulent *K. pneumoniae* (hvKP) were absent, such as the aerobactin synthesis cluster (*iucABCD*), the salmochelin operon (*iroBCDN*), the yersiniabactin system (*ybt*, *fyuA*, *irp*), and the hypermucoviscosity regulators (*rmpA* and *rmpA2*). The presence of only baseline virulence loci, together with the absence of these key hypervirulence genes, indicated that *K. pneumoniae* CGMCC 31459 represents a classical, non-hypervirulent strain. Complementary antimicrobial resistance (AMR) analysis using CARD/RGI and ResFinder revealed a limited set of chromosomally encoded resistance determinants, including *blaSHV-41* (class A β-lactamase), *fosA6* (fosfomycin-inactivating enzyme), and the *oqxAB* efflux operon with its regulator *baeR* (Table S7). No carbapenemase (*blaKPC*, *blaNDM*) or colistin resistance (*mcr*) genes were detected. This virulence–resistance profile reflects intrinsic chromosomal characteristics typical of classical *K. pneumoniae*, lacking both hypervirulent and high-level mobile AMR determinants, thereby supporting its classification as a genetically stable strain with low pathogenic and antimicrobial resistance potential.

Mobile genetic element analysis further showed that *K. pneumoniae* CGMCC 31459 harbors four prophage regions (including one intact prophage of 75.8 kb, Table S8), four genomic islands, and four plasmids. None of the prophages or genomic islands carried detectable virulence or AMR genes. Among the plasmids, only the largest one (199,662 bp) contained a limited number of secretion-system-related genes (*tssH-5/clpV*, *clpV1*, *fleQ*, *algW*), without any hypervirulence or resistance markers (Table S9). These findings suggest that the mobile genetic elements in *K. pneumoniae* CGMCC 31459 primarily contribute to fundamental physiological adaptation and structural stability rather than pathogenicity, consistent with its low-virulence phenotype.

### 3.2. Pan-Genome Analysis of Klebsiella pneumoniae

#### 3.2.1. Genomic Collection of *Klebsiella pneumoniae*

Thirteen *Klebsiella pneumoniae* strains from diverse sources and sequence types were selected for pan-genome analysis and phylogenetic inference, with all genome sequences obtained from the GenBank database. The analyzed strains exhibited genome sizes ranging from 5.07 to 5.40 Mb, GC contents between 56.84% and 57.55%, coding gene counts varying from 4776 to 5572, and contig numbers spanning 1 to 7. Genome completeness and contamination rates were assessed using the CheckM program (Table S10), revealing that all analyzed strain genomes exhibited ≥99.0% completeness with ≤1.0% contamination, meeting the criteria for near-complete (≥90%) and low-contamination (≤5%) genomes, thereby qualifying for downstream analyses.

**Figure 3:**
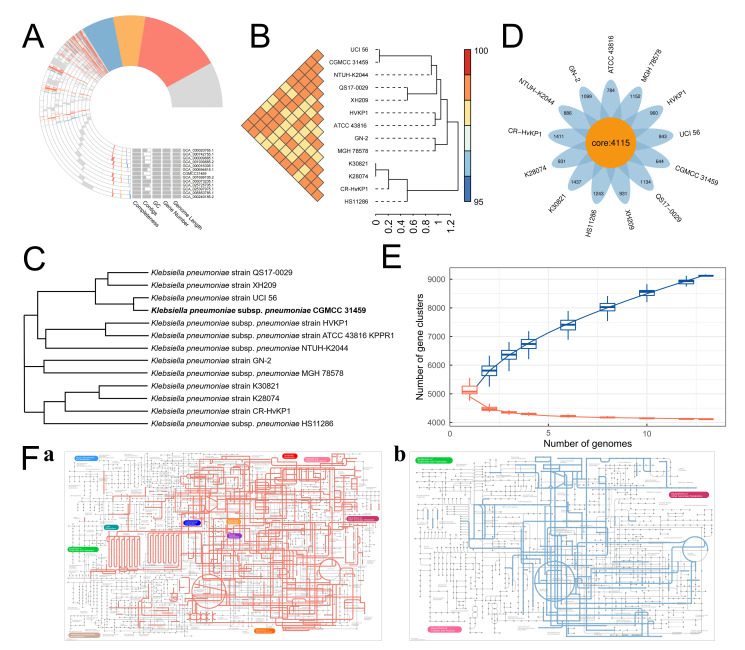
Comparative genomic features and metabolic pathway analysis of *K. pneumoniae* CGMCC 31459: (**A**) COG classification statistics of *K. pneumoniae* strains included in the analysis; (**B**) ANI value comparison; (**C**) Phylogenetic tree of *K. pneumoniae* (the bolded parts indicate the strains used in this study); (**D**) Pan-genome flower plot of *K. pneumoniae* CGMCC 31459 and other analyzed *K. pneumoniae* genomes; (**E**) Pan-genome and core-genome curves of 13 *K. pneumoniae* strains, with orange and blue lines representing the pan-genome and core-genome, respectively; (**F**) Metabolic pathway analysis of *K. pneumoniae* CGMCC 31459: (**a**) Metabolic pathways; (**b**) Biosynthesis of secondary metabolites.

Functional annotation of *K. pneumoniae* CGMCC 31459 demonstrated predominant metabolic functions, encompassing genes related to amino acid, carbohydrate, and lipid metabolism, along with genes encoding efflux pumps (*acrB*, *emrA*) and transporters (*ttuB*), suggesting robust metabolic activity. The COG functional distributions of other *K. pneumoniae* strains were similar, though some exhibited unique characteristics: *K. pneumoniae* HS11286 and *K. pneumoniae* GN-2 carried additional antibiotic resistance genes (*aadB*, *bla*), while *K. pneumoniae* HVKP1 possessed cellulose synthase-related genes that may enhance biofilm formation capability (Figure 3A). Overall, the 13 *K. pneumoniae* strains showed highly conserved genomes with core metabolic similarities, though certain strains may adapt to diverse environmental stresses through specific genes (efflux pumps, biofilm-associated genes).

#### 3.2.2. Phylogenetic Analysis of *Klebsiella pneumoniae*

Average nucleotide identity (ANI) analysis was employed to investigate genomic characteristics of *K. pneumoniae* (Figure 3B). The ANI results demonstrated that *K. pneumoniae* CGMCC 31459 maintained high genomic similarity (ANI > 98%) with all twelve compared *K. pneumoniae* strains, showing the highest identity with *K. pneumoniae* UCI56, suggesting their likely classification within the same subspecies. *K. pneumoniae* CGMCC 31459 exhibited slight but detectable ANI differences with hypervirulent strains NTUH-K2044 and KPPR1, suggesting potential key functional divergences in their genomes. All compared strains showed ANI values above the species demarcation threshold (95%), confirming that all 13 strains, including *K. pneumoniae* CGMCC 31459, belong to the same species.

Phylogenetic analysis based on whole-genome sequences classified the 13 *K. pneumoniae* strains into three major evolutionary clades. *K. pneumoniae* CGMCC 31459 showed the closest phylogenetic relationship with *K. pneumoniae* UCI 56 of the same MLST type, indicating highly similar genomic characteristics between these strains (Figure 3C). *K. pneumoniae* CGMCC 31459 exhibited significant evolutionary divergence from known hypervirulent or clinical isolates (including *K. pneumoniae* QS17-0029 and *K. pneumoniae* NTUH-K2044), suggesting that exopolysaccharide-producing strains may exhibit significant differences in genomic composition or expression patterns compared to pathogenic strains. Its strong exopolysaccharide biosynthesis capacity may substitute for the functions of certain virulence factors. Additionally, *K. pneumoniae* GN-2 and *K. pneumoniae* MGH 78578 together form a highly supported and relatively independent clade, while *K. pneumoniae* CGMCC 31459 shows comparatively greater genetic distance from both, suggesting potentially significant divergence in their genetic characteristics. These evolutionary relationships indicate that *K. pneumoniae* CGMCC 31459 likely shares specific metabolic traits with its phylogenetically similar strain *K. pneumoniae* UCI 56, particularly regarding carbohydrate metabolism-related gene modules.

#### 3.2.3. Pan-Genome and Core-Genome Analysis of *Klebsiella pneumoniae*

Pan-genome and core genome analyses were performed on a total of 13 *Klebsiella pneumoniae* strains, including *K. pneumoniae* CGMCC 31459 and the reference strain *K. pneumoniae* ATCC 43816 (Figure 3D,E). The pan-genome analysis showed that as the number of genomes increased, the number of core gene clusters gradually decreased, while the number of pan gene clusters increased. In total, the 13 genomes contained 9121 pan gene clusters and 4115 core gene clusters, among which *K. pneumoniae* CGMCC 31459 contained 644 accessory genes (Figure 3D). Overall, *K. pneumoniae* exhibits an open and diverse pan-genome structure, undergoing frequent evolutionary changes and adapting to diverse ecological environments through gene gain/loss or horizontal gene transfer [46].

#### 3.2.4. KEGG Pathway Analysis of *Klebsiella pneumoniae* CGMCC 31459

The KEGG Orthology (KO) entries of *K. pneumoniae* CGMCC 31459 were mapped to KEGG pathways. KEGG pathway analysis indicated that *K. pneumoniae* CGMCC 31459 possesses a typical metabolic architecture and contains a relatively complete exopolysaccharide biosynthesis pathway, involving several key steps such as sugar precursor activation, glycosidic bond formation, and polysaccharide polymerization and transport (Figure 3F). Glucose plays a central role in the exopolysaccharide synthesis of *K. pneumoniae* CGMCC 31459. Glucose-1-phosphate adenylyltransferase and UTP-glucose-1-phosphate uridylyltransferase are jointly involved in the synthesis of UDP-glucose, while UDP-glucose 4-epimerase and UDP-glucose 6-dehydrogenase catalyze the formation of UDP-galactose and UDP-glucuronic acid, respectively, providing sugar donors for the incorporation of different monosaccharide units into the polysaccharide structure. Additionally, upstream processes of sugar metabolism, such as glucokinase and glucose-6-phosphate isomerase, are involved in glucose activation and isomerization. Glucosamine-fructose-6-phosphate aminotransferase and phosphoglucosamine mutase participate in the synthesis of amino sugar precursors, further expanding the structural diversity of polysaccharide components. Notably, several structural genes related to O-antigen and exopolysaccharide polymerization and transport were also identified, including Wzy polymerase, WecA transferase, and RfaL ligase, which are responsible for the polymerization of repeating sugar units, glycosyl transfer to lipid carriers, and the final linkage of polysaccharides to the lipopolysaccharide backbone, respectively. The cooperative action of these enzymes suggests that *K. pneumoniae* CGMCC 31459 likely employs a Wzy-dependent polysaccharide synthesis mechanism, completing the synthesis and activation of sugar precursors intracellularly, followed by assembly into complex exopolysaccharide structures via membrane-associated systems. Overall, the carbohydrate metabolism annotation of *K. pneumoniae* CGMCC 31459 covers multiple KEGG pathways, including amino sugar and nucleotide sugar metabolism, starch and sucrose metabolism, and involves the biosynthesis and regulation of various sugar donors, providing abundant metabolic substrates and structural modules for polysaccharide synthesis.

### 3.3. Observation of Bacterial Morphology and Exopolysaccharide Layer in K. pneumoniae CGMCC 31459

**Figure 4:**
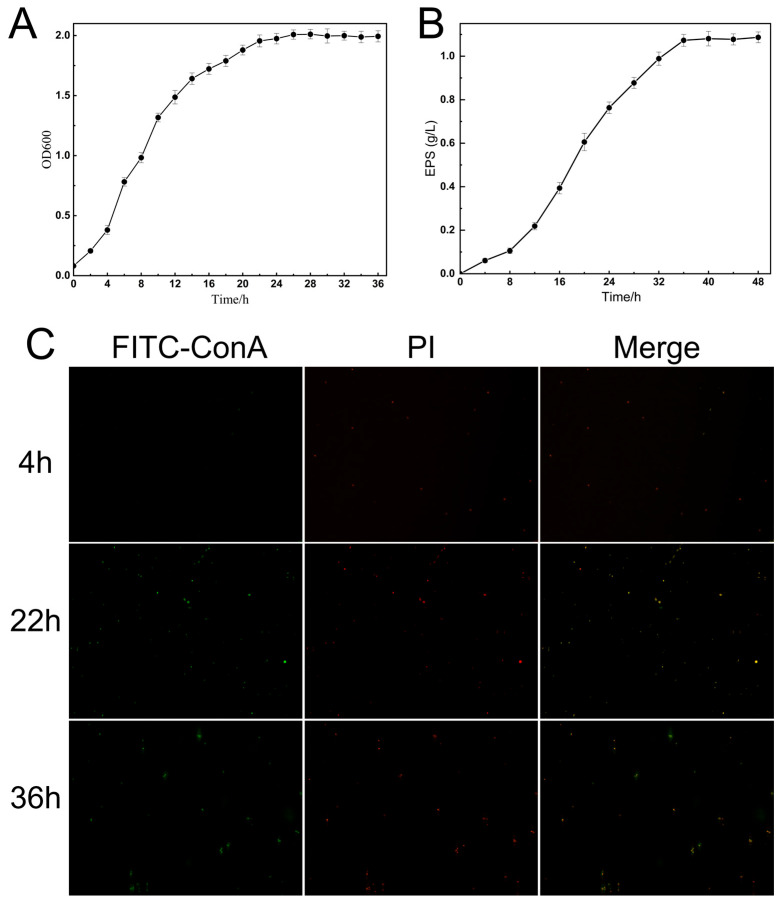
Study on the secretion characteristics of exopolysaccharide in *K. pneumoniae* CGMCC 31459. (**A**) Growth curve of *K. pneumoniae* CGMCC 31459; (**B**) EPS production curve of *K. pneumoniae* CGMCC 31459 over time; (**C**) confocal laser scanning microscopy analysis of *K. pneumoniae* CGMCC 31459 and its EPS, Figure 4C is provided in clear form in the Supplementary Materials.

The growth curve of K. pneumoniae CGMCC 31459 is shown in Figure 4A. The strain entered the logarithmic phase at 4 h and grew rapidly, reaching the late logarithmic phase at 22 h before gradually entering the stationary phase. The exopolysaccharide production of *K. pneumoniae* CGMCC 31459 over time is presented in Figure 4B. The accumulation of exopolysaccharide increased with prolonged cultivation time, peaking at 36 h with minimal subsequent changes. Based on the growth dynamics and exopolysaccharide production of *K. pneumoniae* CGMCC 31459, bacterial samples at 4 h, 22 h, and 36 h were selected for morphological studies.

FITC-ConA can specifically bind to α-mannose and α-glucose residues, thereby fluorescently labeling exopolysaccharides (green fluorescence); meanwhile, PI can penetrate bacterial cells with damaged membrane structures and produce red fluorescence upon binding to DNA. Based on the distinct properties of these two fluorescent probes, effective differentiation between bacterial cells and the exopolysaccharide layer can be achieved (Figure 4C). During the fermentation culture of *K. pneumoniae* CGMCC 31459, prominent red fluorescence with weak green fluorescence was predominantly observed at 4 h, indicating that the bacterial cells were in an active division state with growth predominating and minimal exopolysaccharide secretion. By 22 h, distinct red fluorescence remained clearly visible while the green fluorescence signal intensified significantly, forming a pronounced fluorescent ring-like structure. This demonstrated that *K. pneumoniae* CGMCC 31459 had entered the stationary growth phase and initiated substantial exopolysaccharide secretion, forming a continuous exopolysaccharide layer on the cell surface. At 36 h, the green fluorescence further intensified, forming a uniformly thick and continuous, dense fluorescent ring, with the red fluorescence signal completely encapsulated within, indicating that the mature exopolysaccharide layer had fully covered the bacterial surface. Overall, this dynamic change process visually demonstrates the metabolic shift in *K. pneumoniae* CGMCC 31459 from being dominated by bacterial proliferation to being primarily focused on the synthesis of secondary metabolites represented by EPS.

### 3.4. Structural Characterization of Exopolysaccharides

#### 3.4.1. Analysis of Molecular Weight and Monosaccharide Composition of EPS-KP

**Figure 5:**
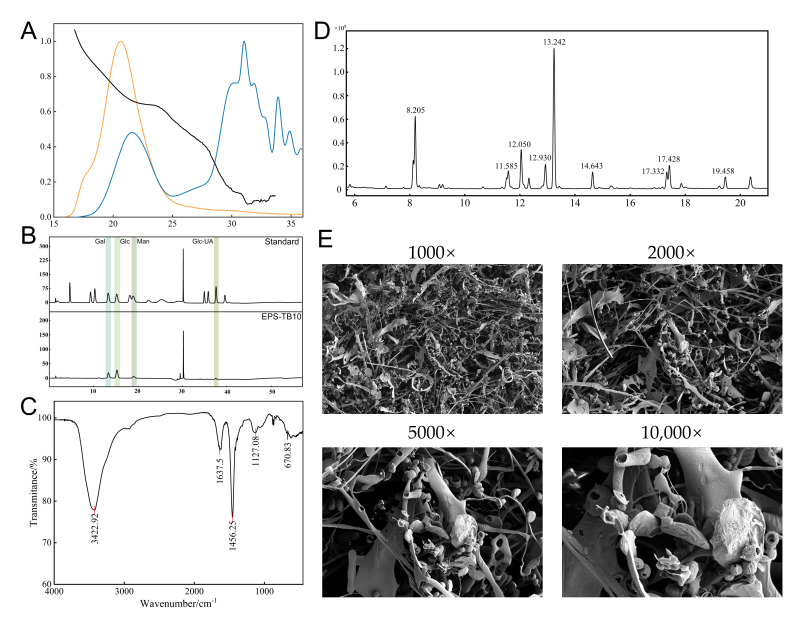
Structural characterization of EPS-KP: (**A**) Molecular weight distribution of EPS-KP; (**B**) Monosaccharide composition of EPS-KP; (**C**) FTIR analysis of EPS-KP; (**D**) Methylation analysis of EPS-KP; (**E**) SEM image of EPS-KP. Note: (**A**) The orange curve represents the multi-angle laser light scattering (MALLS) signal; the blue curve indicates the differential refractive index (dRI) signal, whose response value depends on the refractive index change in the column effluent; the black curve shows the molecular weight derived from fitting these two signals.

The molecular weight distribution and monosaccharide composition of exopolysaccharides are key determinants of their physicochemical properties and bioactivities. The molecular weight of EPS-KP was determined using a gel permeation chromatography-multi-angle laser light scattering-refractive index detection (GPC-MALS-RI) system (Figure 5A). The results showed that EPS-KP had a number-average molecular weight (Mn) of 52.745 kDa, a weight-average molecular weight (Mw) of 503.908 kDa, and a peak molecular weight (Mp) of 23.375 kDa. The polydispersity index (Mw/Mn) was 9.554, indicating an extremely broad molecular weight distribution. This suggests that EPS-KP may be a mixture of components with varying degrees of polymerization or exhibit significant structural heterogeneity. The root-mean-square radii (Rn, Rw, Rz) were 39.756 nm, 37.1 nm, and 32.435 nm, respectively, suggesting that the polysaccharide molecules likely adopted a random coil or near-spherical conformation in solution. Monosaccharide composition analysis was performed to determine the types and quantities of various monosaccharides in the exopolysaccharides. The ion chromatography profile of the acid-hydrolyzed EPS obtained from *K. pneumoniae* CGMCC 31459 fermentation is shown in the corresponding figure, while the detailed monosaccharide composition and ratios are presented in Table S11. EPS-KP was primarily composed of glucose (49.48%), galactose (28.81%), mannose (16.29%), and glucuronic acid (5.42%) (Figure 5B). Neutral sugars accounted for 94.582% of the total sugar content, while uronic acids constituted only 5.418%, indicating that EPS-KP is a weakly acidic heteropolysaccharide dominated by neutral monosaccharides.

#### 3.4.2. FT-IR Spectroscopic Analysis of EPS-KP

The FTIR analysis of EPS-KP revealed its typical polysaccharide structural characteristics (Figure 5C). The broad and strong absorption peak at 3422.92 cm^−1^ was attributed to the stretching vibration of hydroxyl (-OH) groups, associated with the presence of intermolecular or intramolecular hydrogen bonds. This indicates the existence of an extensive hydrogen bond network in the sample, which is a distinctive feature of exopolysaccharide molecules. The medium-intensity absorption peak at 1637.5 cm^−1^ primarily corresponds to the stretching vibration of the C-O bond, while possibly overlapping with the carbonyl (C=O) vibration signals from carboxyl (-COOH) or amide (-CONH-) groups, suggesting the presence of acidic or nitrogen-containing functional groups in EPS-KP. The characteristic peak at 1456.25 cm^−1^ is attributed to the bending vibration of C-H bonds, including the scissoring vibration of methylene (CH_2_) and the symmetric deformation vibration of methyl (CH_3_) groups. The distinct absorption peak at 1127.08 cm^−1^ is attributed to the stretching vibration of C-O-C bonds, serving as a characteristic marker for pyranose ring skeletal vibrations. The intensity of this peak is closely correlated with both the type of substituents on the sugar ring and the configuration of glycosidic bonds. In the low wavenumber region (900–600 cm^−1^), multiple absorption peaks further elucidate the structural characteristics of the sugar rings. The peaks observed at 867.15 cm^−1^ and 621.66 cm^−1^ likely originate from sugar ring skeletal vibrations or out-of-plane bending vibrations of C-O bonds, while the absorption peak at 670.83 cm^−1^ is associated with vibrational modes related to the ring conformation.

#### 3.4.3. Methylation Analysis of EPS-KP

Methylation analysis is a crucial method for elucidating the structural characteristics of polysaccharides. Glucose was identified as the primary monosaccharide component, with 1,4-linked glucose (4-Glc) exhibiting the highest relative molar ratio (35.01%). This indicates that the main chain is predominantly composed of glucose units connected via 1→4 glycosidic bonds. Meanwhile, a portion of the glucose residues were linked via 1→3 glycosidic bonds (3-Glc(p), 10.48%), while others served as branch points in the form of 1,4,6-linked glucose (4,6-Glc(p), 6.44%) and 1,3,4,6-linked glucose (3,4,6-Glc(p), 3.31%), demonstrating that this polysaccharide has a highly branched structure. Galactose was the second major monosaccharide component in EPS-KP, primarily existing as non-reducing terminal residues (t-Gal(p), 16.72%). Additionally, some galactose units were connected via 1→2 linkages (4.65%) and 1→2,6 linkages (4.45%), further contributing to the branching complexity of the polysaccharide. Mannose served as the third predominant monosaccharide constituent in EPS-KP, with its terminal derivative t-Man(p) representing 6.01% of the composition. A portion of mannose residues were connected through 1→3 (2.04%) and 1→4 (6.75%) glycosidic linkages, potentially distributed within both the main chain and side chains of the polysaccharide (Figure 5D). This structural arrangement contributes significantly to the enhanced diversity of the overall molecular architecture. Methylation analysis unveiled the highly branched structure and complex linkage patterns of EPS-KP. The polysaccharide is primarily composed of glucose and galactose, with glucose forming the backbone while galactose mainly exists as non-reducing terminal residues that participate in branch formation. A small proportion of glucuronic acid was detected in the monosaccharide composition, which may contribute to the overall acidic character and hydrophilicity of the polysaccharide. As no carboxyl reduction was performed during methylation, the linkage information for glucuronic acid was not captured, and the structural data presented here mainly reflect the linkage patterns of neutral sugar residues. This complex macromolecular network, characterized by abundant non-reducing terminal units, diverse branching patterns, and a small fraction of acidic components, forms a highly sophisticated and functionally versatile architecture. Such structural complexity fundamentally underpins the polysaccharide’s exceptional properties and stable physicochemical characteristics.

#### 3.4.4. SEM Analysis of EPS-KP

SEM imaging revealed the microstructural characteristics of EPS-KP at varying magnifications (1000×, 2000×, 5000×, and 10,000×) (Figure 5E). At lower magnification levels, EPS-KP exhibited a loose fibrous three-dimensional network with distinct inter-fibrillar crosslinks, forming large pores and an open spatial framework. As the magnification increased, finer fibrous structures became clearly resolved, exhibiting distinct surface porosity, textural features, and corrugated morphology—characteristics indicative of abundant active sites and superior adsorption capacity. Furthermore, high-magnification imaging revealed irregular particulate matter and spherical aggregates, potentially derived from polysaccharide degradation byproducts. These structures could concurrently impart supplementary functional properties to the system. The intricate three-dimensional network architecture and large specific surface area of EPS-KP provide abundant reactive sites for free radical scavenging, while its porous structure and fibrous network characteristics further enhance its capacity for efficient interactions with free radicals.

### 3.5. Analysis of Antioxidant Activity of EPS-KP

**Figure 6:**
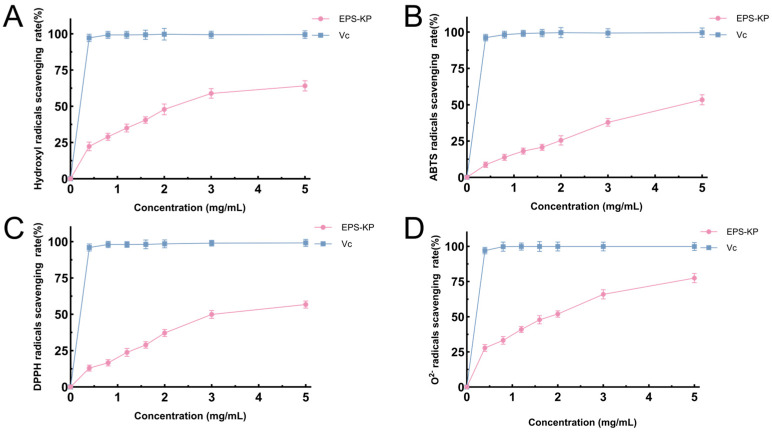
Free radical scavenging activity of EPS-KP:(**A**) Hydroxyl radicals; (**B**) ABTS radicals; (**C**) DPPH radicals; (**D**) Superoxide anion radicals. Ascorbic acid (Vc) was used as a positive control in all assays.

With the deepening research on natural bioactive compounds, exopolysaccharides (EPS) have been found to possess remarkable antioxidant properties and may serve as highly efficient and non-toxic free radical scavengers. This study systematically evaluated the scavenging capacities of exopolysaccharides from *K. pneumoniae* CGMCC 31459 against hydroxyl radicals (-OH), ABTS radicals (ABTS), DPPH radicals (DPPH), and superoxide anion radicals (O_2_^−^) (Figure 6). The experimental results showed that as the concentration increased, the exopolysaccharides from *K. pneumoniae* CGMCC 31459 exhibited significant scavenging activity against all four types of free radicals, with the scavenging rate gradually rising and eventually stabilizing, demonstrating a clear concentration-dependent effect (0.4–5 mg/mL). At the lowest tested concentration (0.4 mg/mL), EPS-KP exhibited scavenging rates of 22.29%, 8.75%, 13.02%, and 27.79% against hydroxyl radicals, ABTS radicals, DPPH radicals, and superoxide anion radicals, respectively. When the concentration increased to 2 mg/mL, the scavenging rates rose to 47.88%, 25.45%, 37.11%, and 51.63%, respectively, and further reached 64.10%, 53.42%, 56.74%, and 77.47% at 5 mg/mL. Based on the concentration-dependent scavenging results, the calculated IC_50_ values were 2.302 mg/mL (hydroxyl radical), 4.683 mg/mL (ABTS), 3.520 mg/mL (DPPH), and 1.634 mg/mL (superoxide anions), respectively, and the corresponding fitting curves are shown in Figure S3. The free radical scavenging efficacy of EPS-KP followed the order: superoxide anion radical > hydroxyl radical > DPPH radical > ABTS radical. Notably, EPS-KP demonstrated the most prominent scavenging effect on superoxide anions, with a scavenging rate exceeding 50% at 2 mg/mL, showing a distinct linear increasing trend with concentration elevation. The hydroxyl radical scavenging activity ranked second, while the relatively lower activities against DPPH and ABTS radicals might be related to the low uronic acid content and limited conjugated electron systems within the EPS-KP structure.

### 3.6. C. elegans Experiments

#### 3.6.1. EPS-KP Significantly Extended the Lifespan of *C. elegans*

**Figure 7:**
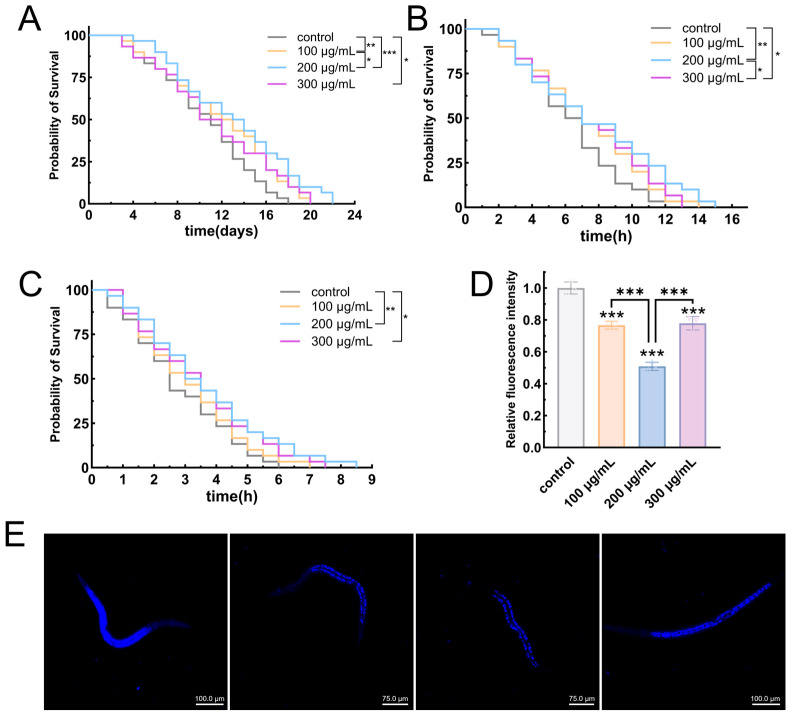
EPS-KP extends the lifespan and improves the healthspan of *C. elegans*: (**A**) Effect of EPS-KP treatment on the lifespan of *C. elegans*; (**B**) Survival curve under heat stress; (**C**) Survival curve under oxidative stress; (**D**) Relative fluorescence intensity of lipofuscin in *C. elegans*; (**E**) Representative fluorescence images of *C. elegans*. * *p* < 0.05, ** *p* < 0.01, *** *p* < 0.001; comparisons between groups are indicated by horizontal bars. Comparisons with the control group are indicated above the columns, while horizontal bars represent comparisons between experimental groups.

Lifespan, a direct indicator of *C. elegans* health status and overall physiological function, serves as a critical parameter for evaluating aging progression and experimental intervention efficacy. In this study, EPS-KP was administered at three concentrations (100 μg/mL, 200 μg/mL, and 300 μg/mL) to assess its anti-aging activity, with an untreated group serving as the control. The experimental results demonstrated that EPS-KP treatment significantly extended the mean lifespan of nematodes in a concentration-dependent manner (Figure 7A, Table 1). Compared with the control group, worms fed with 100 µg/mL, 200 µg/mL and 300 µg/mL EPS-KP showed extended mean lifespans of 11.83 days (*p* < 0.01), 12.89 days (*p* < 0.001) and 10.79 days (*p* < 0.05), representing increases of 20.2% (*p* < 0.01), 30.9% (*p* < 0.001) and 11.6% (*p* < 0.05), respectively. The most significant lifespan extension was observed at 200 μg/mL, with the median lifespan increasing from 9.5 days to 13.5 days and maximum lifespan extending from 17 days to 21 days. All parameters demonstrated optimal effects at this concentration, suggesting it may represent the best bioactive window. The lifespan-extending effect of EPS-KP exhibited a characteristic “inverted U-shaped” dose–response curve. Within the 100–200 μg/mL concentration range, the longevity-promoting effects increased with higher concentrations. However, when the concentration reached 300 μg/mL, the efficacy declined. Comprehensive analysis revealed that EPS-KP demonstrated the most significant anti-aging effects at 200 µg/mL.

#### 3.6.2. EPS-KP Improves Stress Resistance in *C. elegans*

Thermal and oxidative stress have been demonstrated to accelerate cellular oxidation processes in organisms, thereby promoting aging. This study systematically evaluated the protective effects of EPS-KP on *C. elegans* under both oxidative and thermal stress conditions. The experimental results revealed that EPS-KP significantly extended the survival time of nematodes exposed to these stress conditions. Under thermal stress conditions, control group nematodes demonstrated a mean lifespan of 6.34 h, a median lifespan of 6.5 h, and a maximum lifespan of 12 h. Treatment with EPS-KP at concentrations of 100, 200, and 300 μg/mL significantly extended the mean lifespan to 7.22 h (*p* < 0.01), 7.985 h (*p* < 0.001), and 7.31 h (*p* < 0.001), respectively, representing lifespan extensions of 13.9% (*p* < 0.01), 25.9%(*p* < 0.001), and 15.3% (*p* < 0.001) compared to untreated controls (Figure 7B, Table 2). Among the EPS-KP treatment groups, the 200 μg/mL concentration demonstrated optimal protective efficacy. This treatment extended the median survival time (50% survival) from 6.5 h in controls to 7 h under thermal stress conditions, while also significantly prolonging the maximum survival duration. These results clearly indicate that EPS-KP effectively enhances thermotolerance in *C. elegans*. In the oxidative stress assays, untreated control nematodes exhibited a mean survival time of 2.83 h, a median lifespan of 2.75 h, and a maximum lifespan of 5.5 h. Treatment with EPS-KP at concentrations of 100, 200, and 300 μg/mL significantly extended the mean survival time to 3.1 h, 3.69 h (*p* < 0.001), and 3.53 h (*p* < 0.001), representing percentage increases of 9.5%, 30.4% (*p* < 0.001), and 24.7% (*p* < 0.001) compared to the control group, respectively. The median lifespan increased to 3–3.5 h with maximum lifespan reaching 8 h, with the 200 μg/mL treatment group demonstrating the most significant effects (Figure 7C, Table 2). Notably, higher concentrations of EPS-KP provided substantially greater protective effects than lower concentrations. While the 300 μg/mL treatment group showed slightly reduced efficacy compared to the 200 μg/mL group, it still significantly outperformed the 100 μg/mL treatment, conclusively demonstrating EPS-KP’s effectiveness in enhancing oxidative stress resistance in nematodes.

Comprehensive analysis revealed that under both stress conditions, the 200 μg/mL EPS-KP treatment consistently demonstrated optimal protective efficacy, increasing mean survival time by 30.4% (oxidative stress) and 25.9% (thermal stress), respectively. These findings provide crucial experimental evidence for elucidating the stress-resistant molecular mechanisms of EPS-KP and its potential applications. Building upon previous structural characterization and antioxidant activity data of EPS-KP, we hypothesize that specific structural motifs within the EPS-KP molecule may play a pivotal role in activating the antioxidant defense system in *C. elegans*.

#### 3.6.3. EPS-KP Exerts Anti-Aging Effects by Reducing Lipofuscin Accumulation

Lipofuscin is an indigestible fluorescent pigment that accumulates during cellular aging, whose content positively correlates with the organismal aging degree. It is widely recognized as a crucial biomarker for assessing aging progression. This study investigated the effects of EPS-KP at varying concentrations on lipofuscin accumulation in *C. elegans*. The results demonstrated that EPS-KP treatment significantly suppressed lipofuscin accumulation (Figure 7D,E), with the 100 μg/mL dose reducing lipofuscin fluorescence intensity by 23.3% (*p* < 0.001) compared to untreated controls; The 200 μg/mL treatment group exhibited the most pronounced effects, showing a 49.1% (*p* < 0.001) reduction in fluorescence intensity. While the 300 μg/mL group demonstrated a rebound in lipofuscin levels compared to the 200 μg/mL group, it still maintained a statistically significant decrease of 22.1% (*p* < 0.001) relative to untreated controls. These results clearly demonstrate that EPS-KP effectively inhibits aging lipofuscin accumulation in nematodes, with the 200 μg/mL treatment showing particularly outstanding efficacy—reducing lipofuscin accumulation to approximately half of control levels. Lipofuscin, known as the “aging pigment” generated during cellular metabolism, exhibits accumulation levels closely associated with oxidative damage. Our findings demonstrate that EPS-KP significantly reduces lipofuscin content, which correlates well with previous observations of its remarkable antioxidant activity and lifespan-extending effects in *C. elegans*. We propose that EPS-KP likely mitigates oxidative damage through its antioxidant properties, thereby suppressing lipofuscin accumulation—a potential mechanistic basis for its anti-aging effects.

#### 3.6.4. EPS-KP Enhances Antioxidant Capacity in *C. elegans*

**Figure 8:**
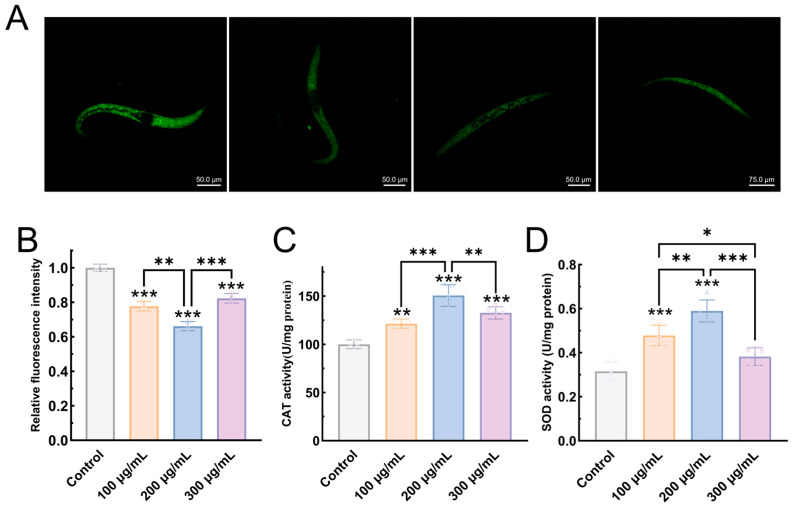
EPS-KP enhances the antioxidant capacity of *C. elegans.* (**A**) Representative fluorescence images of ROS in *C. elegans*; (**B**) Relative fluorescence intensity of ROS in *C. elegans*; (**C**) CAT activity in *C. elegans*; (**D**) SOD activity in *C. elegans*. * *p* < 0.05, ** *p* < 0.01, *** *p* < 0.001; comparisons between groups are indicated by horizontal bars. Comparisons with the control group are indicated above the columns, while horizontal bars represent comparisons between experimental groups.

Reactive Oxygen Species (ROS) represent a group of oxygen-containing reactive molecules whose excessive accumulation triggers oxidative stress, leading to cellular damage. As one of the critical biomarkers for assessing aging in *C. elegans*, ROS levels directly reflect the degree of oxidative damage in organisms. Superoxide dismutase (SOD) and catalase (CAT) serve as two pivotal antioxidant enzymes responsible for ROS scavenging in biological systems. In this study, EPS-KP treatment significantly reduced ROS levels in *C. elegans*. Following EPS-KP administration at various concentrations, the fluorescence intensity of DCFH-DA-labeled nematodes markedly decreased, indicating substantial ROS reduction (Figure 8A,B). Specifically, 100 μg/mL EPS-KP treatment resulted in a significant 22.3% (*p* < 0.001) decrease in ROS levels compared to untreated controls. At the 200 μg/mL concentration, EPS-KP treatment demonstrated optimal ROS-scavenging efficacy, reducing ROS accumulation by 33.9% (*p* < 0.001) compared to untreated controls. In the 300 μg/mL treatment group, ROS levels decreased to 17.7% (*p* < 0.001), showing a modest rebound compared to the 100 μg/mL and 200 μg/mL groups. Reactive oxygen species (ROS) are key mediators of oxidative cellular damage. EPS-KP effectively scavenges ROS in *C. elegans*, with 200 μg/mL showing peak antioxidant activity—consistent with its documented lifespan extension, enhanced stress resistance, and reduced lipofuscin accumulation.

EPS-KP significantly enhances key antioxidant enzyme activities in nematodes, regarding CAT activity (Figure 8C): The control group showed a basal CAT activity of 100.022 U/mg prot. Treatment with 100 μg/mL EPS-KP increased CAT activity to 121.45 U/mg prot (21.4% increase, *p* < 0.01), while the 200 μg/mL group exhibited the highest activity (150.68 U/mg prot, 50.6% increase, *p* < 0.001). At 300 μg/mL, activity slightly declined to 132.63 U/mg prot (32.6% increase, *p* < 0.001). Regarding SOD activity (Figure 8D): EPS-KP treatment significantly enhanced SOD activity in a concentration-dependent manner, with 100, 200, and 300 μg/mL doses increasing the levels to 0.478 U/mg prot (51.7% increase, *p* < 0.001), 0.589 U/mg prot (87.0% increase, *p* < 0.001), and 0.382 U/mg prot (21.3% increase), respectively, compared to the control. Comprehensive analysis revealed that EPS-KP exhibited a similar concentration-dependent activation pattern for both antioxidant enzymes (SOD and CAT): Within the 100–200 μg/mL concentration range, the enzymatic activity increased with rising EPS-KP concentration. The 200 μg/mL treatment group demonstrated optimal activation efficacy. Although the 300 μg/mL group still maintained significant enzyme activation, its effect was notably reduced compared to the 200 μg/mL group. These results align with our previous observations that EPS-KP extends lifespan and enhances stress resistance in nematodes. The findings confirm that EPS-KP effectively strengthens oxidative stress defense by coordinately activating both CAT and SOD antioxidant enzyme systems, thereby prolonging lifespan and exhibiting potent anti-aging activity.

## 4. Discussion

This study focuses on the ST678-type *K. pneumoniae* CGMCC 31459, providing an in-depth analysis of its genomic characteristics, exopolysaccharide (EPS-KP) structure and function, as well as its antioxidant and anti-aging activities. Genomic analysis revealed an abundant carbohydrate-active enzyme system and complex sugar metabolism network, which laid a genetic foundation for its efficient EPS synthesis and modification. Through multi-level structural characterization, it was confirmed that the EPS is a heteropolysaccharide with complex branching architecture. At the functional level, the branched dextran backbone and acidic groups of EPS-KP achieve efficient scavenging of free radicals through a dual mechanism of “physical barrier–antioxidant microenvironment”. In the nematode model, EPS-KP significantly prolonged lifespan and enhanced stress resistance by activating the SOD/CAT enzyme system and inhibiting lipofuscin accumulation. These findings not only provide new insights into bacterial metabolic regulation mechanisms but also open new avenues for developing biosafe anti-aging biologics based on natural polysaccharides.

### 4.1. Genomic Analysis Reveals EPS Biosynthetic Capacity and Environmental Adaptability of K. pneumoniae CGMCC 31459

Through whole-genome analysis of *K. pneumoniae* CGMCC 31459, we systematically characterized its genomic architecture and functional traits, with particular emphasis on evaluating its safety and application potential as a functional EPS-producing strain. This study reveals unique genomic features and environmental adaptation strategies of this bacterial strain. The genome of *K. pneumoniae* CGMCC 31459 consists of a single chromosome and four plasmids. Characterized by high gene density and GC content, these genomic features not only enhance chromosomal stability but may also optimize gene expression efficiency, conferring rapid environmental responsiveness. Functional annotation revealed an abundance of carbohydrate-active enzymes in *K. pneumoniae* CGMCC 31459, including 69 glycoside hydrolases and 33 glycosyltransferases, which collectively provide the molecular basis for EPS biosynthesis, modification, and degradation [3,47]. Notably, the synergistic action of carbohydrate esterases and auxiliary activity enzymes suggests potential complex structural modifications and antioxidant capabilities in its EPS, consistent with experimentally observed bioactivities. Its conserved energy metabolism genes (TCA cycle and glycolysis) and abundant polysaccharide metabolic pathways support the utilization of environmental carbon sources, while the relatively limited number of genes involved in secondary metabolite synthesis and the low potential for polyketide and non-ribosomal peptide production explain, at the molecular level, the strain’s high efficiency in EPS production.

From an evolutionary perspective, the ST678 typing results of *K. pneumoniae* CGMCC 31459 reveal its genetic relationships with other strains. Compared to other hypervirulent or drug-resistant strains, the abundance of carbohydrate metabolism-related genes in *K. pneumoniae* CGMCC 31459 suggests its evolutionary strategy may prioritize metabolic adaptability over pathogenicity. This enables efficient resource competition in nutrient-rich environments while reducing host dependence, consequently lowering the risk of host immune clearance [7,48]. Genomic characterization also supported its biosafety and low virulence potential. The strain lacks major virulence determinants typically associated with hvKP, such as *rmpA*/*rmpA2* and *iucABCD-iutA*, which regulate capsule formation and siderophore-mediated iron uptake. Moreover, classical siderophore clusters (*ybt*, *clb*, *irp*, *ent*) were incomplete, further indicating limited pathogenic potential. The antimicrobial resistance profile revealed only a few intrinsic chromosomal genes without plasmid-borne multidrug resistance islands or carbapenemase genes. These findings collectively suggest that *K. pneumoniae* CGMCC 31459 possesses a non-hypervirulent and low-risk genomic background, supporting its suitability as a safe EPS-producing strain under controlled laboratory and fermentation conditions. Meanwhile, the evolutionary relationships of *K. pneumoniae* CGMCC 31459 further elucidate its genetic diversity and evolutionary position within the species. Its closest phylogenetic affinity with *K. pneumoniae* UCI 56 suggests shared metabolic characteristics and environmental adaptability between these strains. Furthermore, the open and diverse pan-genome of *K. pneumoniae* demonstrates the species’ capacity to continuously acquire novel genes during evolution, enabling adaptation to diverse ecological environments [46,49]. The 39.8% proportion of accessory genes substantiates its evolutionary strategy of rapidly acquiring novel functional genes through horizontal gene transfer, enabling strains to maintain relatively conserved core genomes while expanding their metabolic versatility and ecological niches through acquisition of new genetic modules [49].

*K. pneumoniae* CGMCC 31459 exhibits characteristic environmentally adapted genomic features: it possesses both an efficient carbohydrate metabolic network and a complete EPS biosynthesis-secretion system, demonstrating robust metabolic adaptability, while simultaneously ensuring genetic safety through virulence gene depletion, conserved secretion systems, and streamlined plasmid composition. This precise genotype–phenotype correspondence establishes it as an ideal EPS-producing engineering strain combining production efficiency with application safety, providing a theoretical foundation for developing bioactive substances with dual functional and safety advantages.

### 4.2. Structure–Activity Relationship of EPS-KP: Structural Characteristics Drive Antioxidant and Anti-Aging Functions

EPS-KP is a high-molecular-weight heteropolysaccharide with demonstrates exceptional antioxidant and anti-aging activities. Its biological functions are intrinsically associated with its molecular structure. The high neutral sugar content confers excellent water solubility and stability, while the limited uronic acid content enhances biocompatibility, facilitates metal ion chelation, and suppresses Fenton-reaction-induced free radical generation, thereby further improving antioxidant capacity [50]. EPS-KP exhibits a weight-average molecular weight of 503.9 kDa with broad dispersity, suggesting either a mixture of varying polymerization degrees or significant structural heterogeneity. The high-molecular-weight fractions contribute to stable physical network formation, while the low-molecular-weight components likely enhance bioaccessibility [51,52]. EPS-KP features a backbone structure composed of 1,4-linked glucose residues as the skeletal framework, while incorporating 1,3- and 1,6-linked galactose branching points to form a highly branched three-dimensional network, resulting in a composite architecture characterized by a “rigid skeleton + flexible side chains” configuration. This three-dimensional network not only enhances molecular flexibility and stability but also exposes abundant free hydroxyl groups, providing critical sites for free radical scavenging and antioxidant activity. While our structural analysis has comprehensively delineated the neutral sugar framework, the specific integration pattern of glucuronic acid residues within the EPS-KP architecture warrants further investigation. Monosaccharide composition analysis confirmed the presence of glucuronic acid. However, as the methylation analysis was primarily focused on elucidating the linkages within the neutral sugar backbone, it did not allow for the precise determination of uronic acid linkage patterns. Consequently, the exact positioning of these acidic components within the three-dimensional matrix remains to be fully elucidated. Further investigation employing specialized techniques would be valuable to precisely map the incorporation of glucuronic acid residues, thereby enabling a more complete understanding of the structure–activity relationships in this biologically active polysaccharide.

EPS-KP demonstrates concentration-dependent antioxidant capacity in free radical scavenging assays, exhibiting particularly strong efficacy against superoxide anions and hydroxyl radicals. This superior scavenging capability originates from its unique spatial configuration and strategic distribution of active functional groups [2]. The 1,4-linked backbone combined with 1,3-linked conformations induces random coil formation, exposing hydroxyl groups to the solvent interface and thereby increasing their reactivity with free radicals. Concurrently, the branched architecture significantly elevates reactive site density. The high-molecular-weight fractions further enhance overall stability through intermolecular hydrogen bonding. The predominant peak of relatively low-molecular-weight fractions facilitates diffusion, thereby enhancing bioavailability. The synergistic interplay among distinct structural units endows EPS-KP with dual advantages of antioxidative structural stability and enhanced reactivity [4,44,53]. However, when IC_50_ values were calculated and compared with Vc, the scavenging rates of EPS-KP were found to be at a moderate level, indicating that its antioxidant capacity, though evident, was not excessively strong. This moderate activity can be rationalized by several structural factors. First, the neutral sugar predominance (over 94%) limits the abundance of carboxyl or carbonyl groups that typically enhance electron transfer and metal ion chelation. Second, although a small fraction of glucuronic acid (5.42%) confers weak acidity and certain chelating capability, the relatively low uronic acid content constrains the formation of strong redox-active sites. Third, the high degree of branching and non-reducing termini—while favorable for solubility and dispersion—can spatially hinder electron delocalization across the polysaccharide network and, together with the dense hydrogen-bonding framework, stabilize the molecular conformation but restrict its structural flexibility, thereby leading to a moderate reaction rate with free radicals.

EPS-KP demonstrates remarkable anti-aging effects in the model organism *C. elegans*. At a concentration of 200 μg/mL, the nematodes showed an extended mean lifespan to 12.89 days (representing a 30.9% increase compared to controls), with median and maximum lifespans elevated to 13.5 days and 21 days, respectively, while also demonstrating significantly enhanced tolerance to both thermal and oxidative stress. Meanwhile, EPS-KP significantly reduces ROS levels, suppresses lipofuscin accumulation, and enhances the activities of antioxidant enzymes SOD and CAT in *C. elegans*, demonstrating a dual antioxidative mechanism combining “exogenous scavenging + endogenous activation”. Similarly to EPS-KP, numerous microbial and natural polysaccharides have been reported to exert anti-aging effects through comparable structure–function relationships. The exopolysaccharide from *Lactobacillus plantarum* HY7714 is a heteropolysaccharide composed mainly of glucose and galactose with a branched configuration, which enhances antioxidant enzyme activity and reduces ROS accumulation [54]. Likewise, the EPS from *Levilactobacillus brevis* MKAK9, consisting of repeating units of glucose and rhamnose, exhibited lifespan-extending effects in *C. elegans* that were associated with activation of the SKN-1/Nrf2 antioxidant pathway and upregulation of genes involved in detoxification and stress resistance [55]. In addition to their anti-aging capacity, bacterial polysaccharides have been shown to enhance stress resistance in nematodes. *Lactiplantibacillus plantarum* DS1800 significantly improved *C. elegans* survival under thermal stress and pathogen challenge, demonstrating enhanced stress tolerance alongside longevity [56]. Mechanistic studies of *Bacillus subtilis* biofilms revealed that EPS-mediated matrix formation confers thermotolerance and oxidative stress resistance in worms, accompanied by induction of stress-response genes such as mtl-1 and hsp-70 [57]. These findings are consistent with our observations that EPS-KP not only reduces ROS and lipofuscin accumulation but also enhances endogenous antioxidant enzyme activities and increases tolerance to thermal and oxidative stress. Structure–function correlation analysis revealed that these multi-level anti-aging effects are closely associated with the densely distributed hydroxyl network in its branched architecture, which not only directly neutralizes free radicals but may also maintain redox homeostasis by modulating endogenous antioxidant pathways such as Keap1/Nrf2 [58,59]. Furthermore, its anti-aging activity exhibited a “U-shaped” concentration-dependent response, demonstrating optimal efficacy at 200 μg/mL. This phenomenon primarily results from the formation of a stable three-dimensional network structure at this concentration, which simultaneously ensures maximal exposure of active groups and achieves an optimal balance between molecular diffusion rates and free radical scavenging reactions. At elevated concentrations, excessive molecular aggregation induces both active site occlusion and osmotic stress, while potentially triggering negative feedback regulation of antioxidant pathways, consequently attenuating bioefficacy. These findings highlight the necessity for precise dosage optimization in practical applications to prevent physiological interference induced by high concentrations [19,44,60].

EPS-KP achieves its exceptional antioxidant and anti-aging functions through a three-dimensional structural foundation characterized by “high-molecular-weight backbone scaffolding + branch cluster enhancement + active group empowerment.” This structure–function synergy provides crucial theoretical support for the rational design and bioactivity optimization of functional polysaccharides. Given its excellent biocompatibility and antioxidant potential, EPS from *K. pneumoniae* CGMCC 31459 demonstrates broad application prospects in pharmaceutical and biomaterial fields. Future studies should further explore its immunomodulatory mechanisms and therapeutic potential in specific disease models to facilitate practical applications.

### 4.3. Structural–Functional Insights and Safe Application of EPS-KP

EPSs, as essential metabolic products enabling bacterial adaptation in complex environments, have attracted widespread attention in recent years across multiple domains including functional foods, pharmaceuticals, cosmetics, and environmental remediation, owing to their structural diversity and functional multifunctionality [1,61,62]. The EPS from *K. pneumoniae* CGMCC 31459 demonstrates multi-level synergistic antioxidant and anti-aging properties, providing novel perspectives for research on natural bioactive compounds. EPS-KP not only exhibits potential anti-aging efficacy but may also participate in modulating host immune responses and ameliorating the progression of oxidative stress-related chronic diseases [11,20]. EPS-KP possesses extensive application prospects, with future research focusing on precise structure–function elucidation to thoroughly reveal the relationships between critical glycosidic bonds, branching patterns and corresponding bioactivities, thereby achieving directed design with “controllable structures and tunable functions” and providing theoretical support. Overall, *K. pneumoniae* CGMCC 31459 represents a promising and biosafe EPS-producing strain, expected to serve multiple cutting-edge fields, including sustainable agriculture and environmental remediation, facilitating its transition from laboratory research to market applications and enabling high-value-added transformation.

*K. pneumoniae* CGMCC 31459 lacks key hypervirulence-associated genes and critical resistance markers and shows a genetic background markedly distinct from clinical hvKP isolates; its biosafety risk cannot be considered zero. First, as a member of the *Klebsiella* genus, the strain retains theoretical potential for horizontal gene transfer, particularly in environmental or production waste settings. Second, in large-scale fermentation or industrial settings, risks of bacterial cell carry-over or improper inactivation of waste or residues may pose unintended exposure. To address these potential risks, the following strategies can be adopted. Genomic engineering: employ targeted gene deletions or silencing of residual virulence regulatory genes and non-essential resistance determinants; remove or disable mobile genetic elements (transposons, prophage fragments) to genetically attenuate the strain [63]. Notably, *rmpA* knockout has been shown to significantly reduce capsule formation and hypermucoid phenotype in *K. pneumoniae* [63,64]. Strict biosafety management: perform all cultivation and handling under Biosafety Level-2 or higher conditions, and ensure complete inactivation of bacterial cells in downstream processing and waste disposal. Environmental release control and monitoring: establish surveillance of virulence and resistance gene presence in residual waste or effluent; evaluate survival and persistence of the strain outside laboratory settings to assess real-world risk. By adopting these strategies, it is possible to preserve the EPS production capabilities of *K. pneumoniae* CGMCC 31459 while minimizing biosafety and sanitary risks, thereby supporting its safe use in functional biomaterial applications.

This study systematically investigated the genomic characteristics of ST678-type *K. pneumoniae* CGMCC 31459 and the structural properties/anti-aging mechanisms of its exopolysaccharide EPS-KP, providing crucial theoretical foundations and practical references for developing novel natural antioxidants. Importantly, the integration of genome-based biosafety assessment and genetic attenuation strategies establishes a framework for developing safe, controllable, and industrially applicable EPS-producing Klebsiella strains. However, certain limitations remain: the functions of some genes have not been experimentally validated, and the molecular mechanisms underlying the structure–activity relationship of the polysaccharide are not yet fully elucidated. Future studies will focus on experimental verification of gene functions, investigation of EPS-KP mechanisms of action, and evaluation of its applications across different models, aiming to establish a more robust scientific foundation for the development of biosafe, high-performance microbial cell factories for sustainable production of bioactive polysaccharides.

## 5. Conclusions

*K. pneumoniae* CGMCC 31459, as an ST678-type strain, possesses a rich repertoire of carbohydrate-active enzymes and a complex sugar metabolism network while remaining evolutionarily distant from highly pathogenic strains. EPS-KP is a weakly acidic, highly branched heteropolysaccharide that exhibits strong antioxidant and anti-aging activities, with a superoxide anion scavenging rate of 77.47% (5 mg/mL) and optimal anti-aging effects at a final concentration of 200 µg/mL, extending nematode lifespan by 30.9%. EPS-KP significantly enhances stress resistance in nematodes and increases the activities of the antioxidant enzymes SOD and CAT, effectively prolonging lifespan. These findings provide theoretical and technical support for the application of natural polysaccharides.

## Figures and Tables

**Table 1:** Summary of all lifespan experiments shown in this study.

Experiment Project	Group	Mean Lifespan (Days)	% of Control	Median Lifespan (Days)	MAX Lifespan (Days)
Lifetime Experiment	control	9.84 ± 0.598	100	9.5	17
	EPS-KP-100 µg/mL	11.83 ± 0.318 ^b^	120.2 ^b^	12	19
	EPS-KP-200 µg/mL	12.89 ± 0.366 ^c^	130.9 ^c^	13.5	21
	EPS-KP-300 µg/mL	10.79 ± 0.582 ^a^	116.1 ^a^	11	19

^a^ *p* < 0.05.; ^b^ *p* < 0.01.; ^c^ *p* < 0.001.

**Table 2:** Summary of heat stress and oxidative stress experiments shown in this study.

Experiment Project	Group	Mean Lifespan (h)	% of Control	Median Lifespan (h)	MAX Lifespan (h)
Heat stress	control	6.34 ± 0.069	100	6.5	12
	EPS-KP-100 µg/mL	7.22 ± 0.302 ^b^	113.9 ^b^	7	13
	EPS-KP-200 µg/mL	7.98 ± 0.25 ^c^	125.9 ^c^	7	14
	EPS-KP-300 µg/mL	7.31 ± 0.051 ^c^	115.3 ^c^	7	12
Oxidative stress	control	2.83 ± 0.200	100	2.75	5.5
	EPS-KP-100 µg/mL	3.1 ± 0.058	109.5	3	6.5
	EPS-KP-200 µg/mL	3.69 ± 0.035 ^c^	130.4 ^c^	3.5	8
	EPS-KP-300 µg/mL	3.53 ± 0.13 ^c^	124.7 ^c^	3.5	7

^b^ *p* < 0.01.; ^c^ *p* < 0.001.

## Data Availability

The experiments and experimental results covered in this study are displayed in the article and in the Supplementary Materials; the data presented in this study are available on request from the corresponding author.

## Supplementary Materials

The following supporting information can be downloaded at: https://www.mdpi.com/article/10.3390/biom15111569/s1. Figure S1: Bacterial eggNOG (COG) functional classification chart; Figure S2: Confocal laser scanning microscopy analysis of *K. pneumoniae* CGMCC 31459 and its EPS; Figure S3: Fitted Curve and IC_50_ of Free Radical Scavenging Activity of EPS-KP; Table S1: Sequencing Method of *Klebsiella pneumoniae* CGMCC 31459; Table S2: Statistics of Open Reading Frame Prediction Data; Table S3: Statistical Table of Non-Coding RNA Prediction Data; Table S4: Information on 7 Housekeeping Genes in the *Klebsiella pneumoniae* CGMCC 31459 Whole Genome Sequence; Table S5: Statistical Table of Subcellular Localization Prediction for Protein-Coding Genes; Table S6: Virulence factors identified in *K. pneumoniae* CGMCC 31459; Table S7: Antimicrobial resistance genes detected in *K. pneumoniae* CGMCC 31459 (CARD/RGI & ResFinder); Table S8: Prophage regions predicted in *K. pneumoniae* CGMCC 31459; Table S9: Plasmids 1 identified in *K. pneumoniae* CGMCC 31459; Table S10: General Characteristics of the Genome Sequences Used in This Study; Table S11: Monosaccharide Composition of EPS-KP.

## Abbreviations

The following abbreviations are used in this manuscript:

EPS	Exopolysaccharides
EPS-KP	Exopolysaccharides of *Klebsiella pneumoniae* CGMCC 31459
FTIR	Fourier Transform Infrared Spectroscopy
SEM	Scanning electron microscopy
HPLC	High-performance liquid chromatography
SOD	Superoxide dismutase
CAT	Catalase
FITC-ConA	FITC-labeled Concanavalin A
PI	Propidium Iodide
