# Supplementary material for: Tea-Residue-Derived Klebsiella pneumoniae CGMCC 31459: Genomic Insights and Antioxidant Activity of Its Exopolysaccharides
Source: Biomolecules. 2025 Nov 7;15(11):1569. doi: 10.3390/biom15111569 (PMC12650511; doi:10.3390/biom15111569)
Supplement: Supplementary file 1 [file biomolecules-15-01569-s001.zip › biomolecules-3860600-supplementary.pdf]

Article

Tea Residue-Derived *Klebsiella pneumoniae* CGMCC 31459: Genomic Insights and Antioxidant Activity of Its Exopolysac-charides

Yuanyuan Wang [1], Shengbo Shi [1] , Mingchun Lin [1], Gangrui Zhang [1], Longyu Fang [1], Jinghua Li [1], Rui Geng [1], Yuanxue Zheng [2] and Lujiang Hao [1,*]

[1] School of Bioengineering, Qilu University of Technology (Shandong Academy of Sciences), Jinan 250353, China; 10431230832@stu.qlu.edu.cn (Y.W.); 10431230730@stu.qlu.edu.cn (S.S.); 10431240798@stu.qlu.edu.cn (M.L.); 10431221312@stu.qlu.edu.cn (G.Z.) ; 10431230825@stu.qlu.edu.cn (L.F.); 10431230807@stu.qlu.edu.cn (J.L.); 10431230774@stu.qlu.edu.cn (R.G.); lujiang_hao@qlu.edu.cn (L.H.)

[2] Jinan Qiurong Biotechnology Co., Ltd, Jinan 250353, China; dynwms@126.com(Y.Z.)

* Correspondence: lujiang_hao@qlu.edu.cn (L.H.)

Supplementary Materials: Figure S1: Bacterial eggNOG (COG) functional classification chart; Figure S2: nctional classification chart; Figure S3: Fitted Curve and IC_{50} of Free Radical Scavenging Activity of EPS-KP; Table S1: Sequencing Method of *Klebsiella pneumoniae* CGMCC 31459; Table S2: Statistics of Open Reading Frame Prediction Data; Table S3: Statistical Table of Non-Coding RNA Prediction Data; Table S4: Information on 7 Housekeeping Genes in the *Klebsiella pneumoniae* CGMCC 31459 Whole Genome Sequence; Table S5: Statistical Table of Subcellular Localization Prediction for Protein-Coding Genes; Table S6: Virulence factors identified in *K. pneumoniae* CGMCC 31459; Table S7 Antimicrobial resistance genes detected in *K. pneumoniae* CGMCC 31459 (CARD/RGI & ResFinder); Table S8 Prophage regions predicted in *K. pneumoniae* CGMCC 31459; Table S9 Plasmids 1 identified in *K. pneumoniae* CGMCC 31459; Table S10: General Characteristics of the Genome Sequences Used in This Study; Table S11: Monosaccharide Composition of EPS-KP.

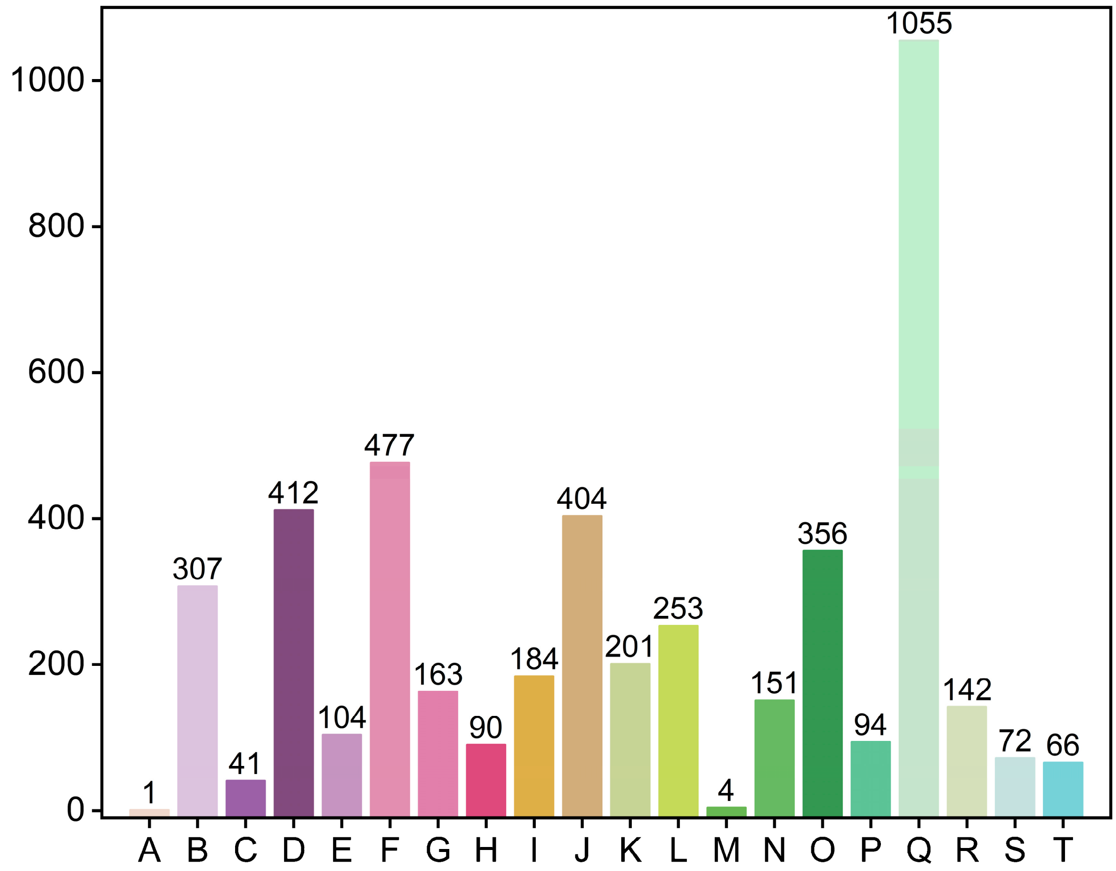

Figure S1: Bacterial eggNOG (COG) functional classification chart. A:RNA processing and modification; B:Energy production and conversion; C:Cell cycle control, cell division, chromosome partitioning; D:Amino acid transport and metabolism; E:Nucleotide transport and metabolism; F:Carbohydrate transport and metabolism; G:Coenzyme transport and metabolism; H:Lipid transport and metabolism; I:Translation, ribosomal structure and biogenesis; J:Transcription; K:Replication, recombination and repair; L:Cell wall/membrane/envelope biogenesis; M:Cell motility; N:Posttranslational modification, protein turnover, chaperones O:Inorganic ion transport and metabolism; P:Secondary metabolites biosynthesis, transport and catabolism; Q:Function unknown; R:Signal transduction mechanisms; S:Intracellular trafficking, secretion, and vesicular transport; T:Defense mechanisms

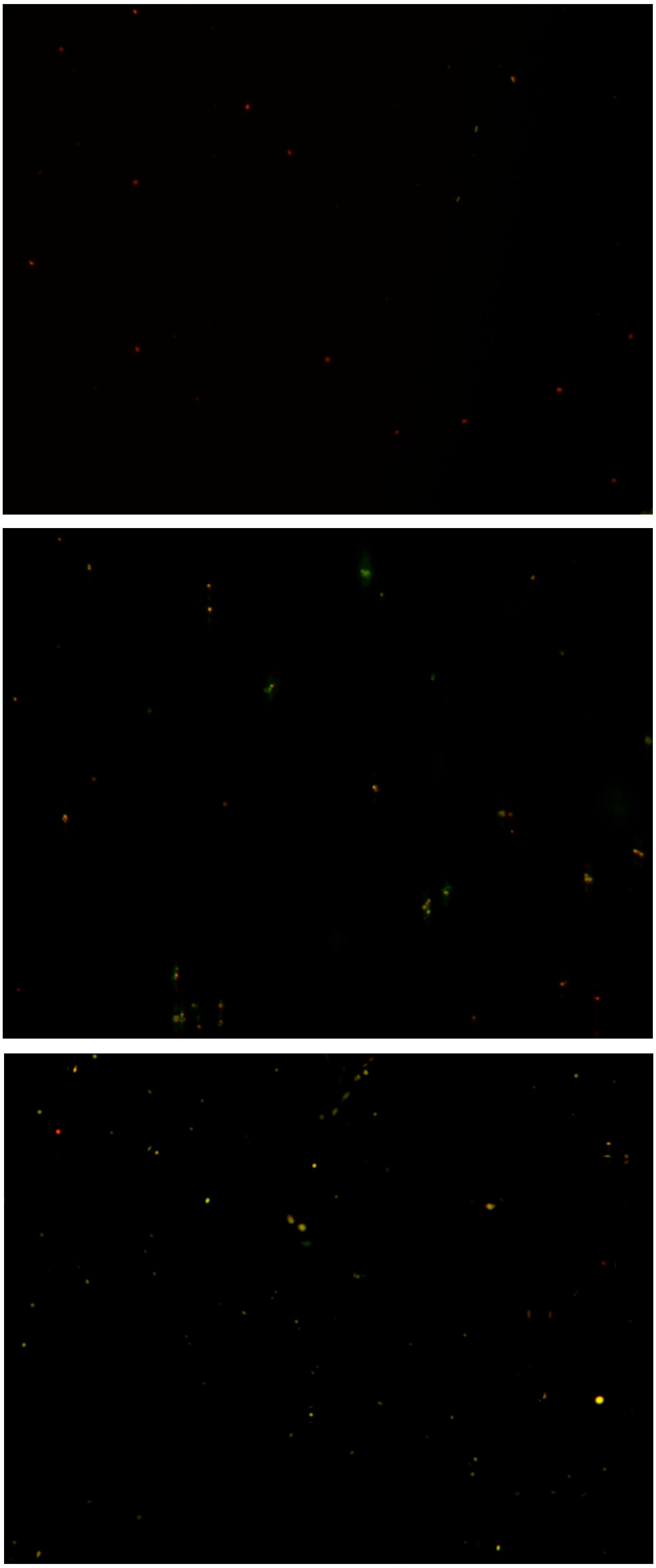

Figure S2: Confocal laser scanning microscopy analysis of *K. pneumoniae* CGMCC 31459 and its EPS. From top to bottom are the merged images at 4 h, 22 h, and 36 h, respectively.

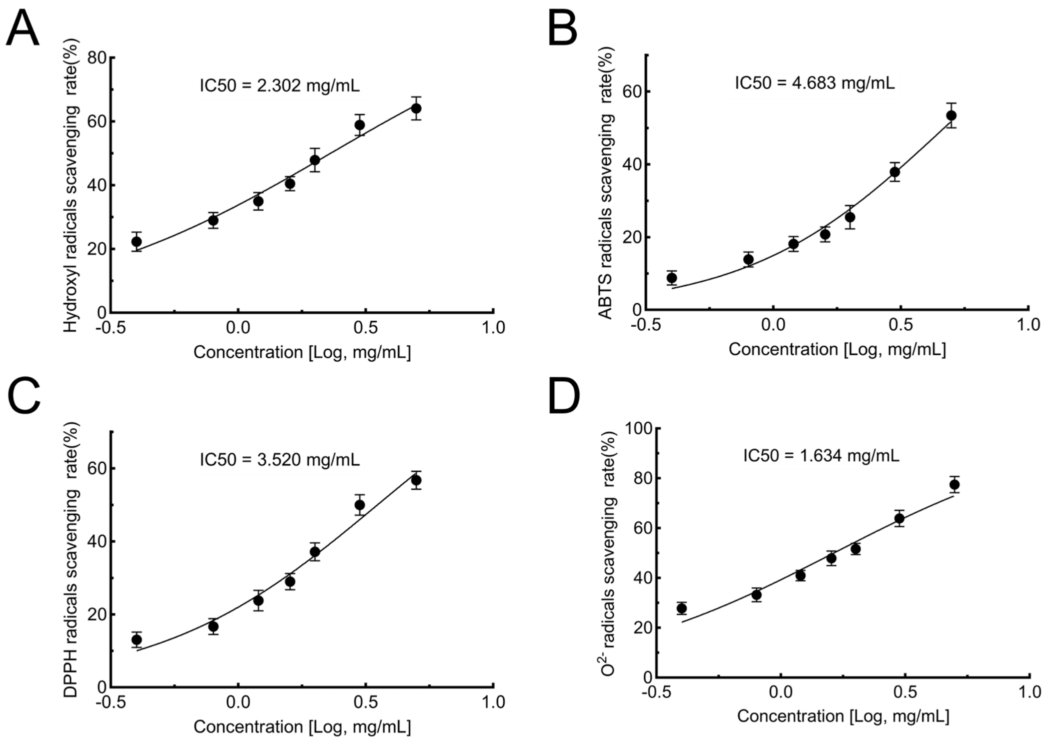

Figure S3: Fitted Curve and IC_{50} of Free Radical Scavenging Activity of EPS-KP. A:Hydroxyl; B:ABTS; C:DPPH; D:O^{2-}

Table S1 Sequencing Method of *Klebsiella pneumoniae* CGMCC 31459

Sample	Lib.Name	Lib.Insert Size	Sequencing platform	Sequencing Mode
K. pneumoniae CGMCC 31459	--	--	ONT	Standard
	PE	400bp	Illumina NovaSeq	Paired-end,2×150bp

Table S2 Statistics of Open Reading Frame Prediction Data

Seq ID	Property	Value
Chr	ORF num	4848
	ORF total length(bp)	4486524 bp
	ORF density(genes/kb)	0.933 genes per kb
	Longest ORF length(bp)	4950 bp
	ORF average length(bp)	925.44 bp
	Intergenetic region length(bp)	708782 bp
	ORF/Genome(coding percentage)%	86.36%

	GC content in ORF region%	58.99%
Plasmid1	ORF num	221
	ORF total length	165225 bp
	ORF density	1.107 genes per kb
	Longest ORF length	5736 bp
	ORF average length	747.62 bp
	Intergenetic region length	34437 bp
	ORF/Genome(coding percentage)	82.75%
	GC content in ORF region	53.80%
Plasmid2	ORF num	4
	ORF total length	2028 bp
	ORF density	0.798 genes per kb
	Longest ORF length	708 bp
	ORF average length	507.00 bp
	Intergenetic region length	2982 bp
	ORF/Genome(coding percentage)	40.48%
	GC content in ORF region	38.07%
Plasmid3	ORF num	4
	ORF total length	1686 bp
	ORF density	0.901 genes per kb
	Longest ORF length	921 bp
	ORF average length	421.50 bp
	Intergenetic region length	2753 bp
	ORF/Genome(coding percentage)	37.98%
	GC content in ORF region	40.98%
Plasmid4	ORF num	2
	ORF total length	633 bp
	ORF density	0.920 genes per kb
	Longest ORF length	405 bp
	ORF average length	316.50 bp
	Intergenetic region length	1540 bp
	ORF/Genome(coding percentage)	29.13%
	GC content in ORF region	45.02%

Table S3 Statistical Table of Non-Coding RNA Prediction Data

Seq ID	Type	Copy Number	Avg. length (bp)	Total length (bp)	percent of genome (%)
chr	5S rRNA	9	110	998	0.0192
	16S rRNA	8	1,536	12,288	0.2365
	23S rRNA	8	2,899	23,192	0.4464
	tRNA	86	78	6,746	0.1298
	ncRNA	116	135	15,726	0.3027

Table S4 Information on 7 Housekeeping Genes in the TB10 Whole Genome Sequence

Locus	Allele	Length	Contig	Start position	End position
gapA	2	450	chr	3058680	3059129
infB	3	318	chr	562015	562332
mdh	1	477	chr	495851	496327
pgi	1	432	chr	4840379	4840810
phoE	109	420	chr	4049736	4050155
rpoB	56	501	chr	4899936	4900436
tonB	18	414	chr	2035643	2036056

Table S5 Statistical Table of Subcellular Localization Prediction for Protein-Coding Genes

Seq ID	Number of Protein-Coding Genes		
	Signal Peptide	Presence of at Least One Transmembrane Helix Region	Secreted Protein Sequence
Chr 1	386	1160	305
Plasmid 1	9	4	8
Plasmid 2	0	1	0
Plasmid 3	0	0	0
Plasmid 4	0	1	0

Table S6 Virulence factors identified in *K. pneumoniae* CGMCC 31459

VFclass	Virulence factors	Related genes	*Klebsiella pneumoniae* CGMCC31459
			CP185868.1
Adherence	Type 3 fimbriae	mrkA	orf00822
		mrkB	orf00823
		mrkC	orf00824
		mrkD	orf00825
		mrkF	orf00826
		mrkH	orf00829
		mrkI	orf00828
		mrkJ	orf00827
	Type I fimbriae	fimA	orf00814
		fimB	orf00817
		fimC	orf00812
		fimD	orf00811; orf04479
		fimE	orf00816
		fimF	orf00810
		fimG	orf00809
		fimH	orf00808
		fimI	orf00813
		fimK	orf00807
Antiphagocytosis	Capsule	-	orf01594; orf01595; orf01596; orf01597; orf01598; orf01599; orf01601; orf01606; orf01607; orf04749
Efflux pump	AcrAB	acrA	orf03712
		acrB	orf00442; orf03713
Iron uptake	Aerobactin	iutA	orf03037
	Ent siderophore	entA	orf03521
		entB	orf03522
		entC	orf03524
		entD	orf03534
		entE	orf03523
		entF	orf03530
		entS	orf03526
		fepA	orf03533; orf04573
		fepB	orf03525
		fepC	orf03529
		fepD	orf03527
		fepG	orf03528
		fes	orf03532

VFclass	Virulence factors	Related genes	*Klebsiella pneumoniae* CGMCC31459
			CP185868.1
	Salmochelin	iroE	orf02437
		iroN	orf02885
Regulation	RcsAB	rcsA	orf01690
		rcsB	orf01471
Secretion system	T6SS-I	-	orf02726
		-	orf02721
		-	orf02720
		clpV/tssH	orf02728
		dotU/tssL	orf02731
		hcp/tssD	orf01354; orf02729
		icmF/tssM	orf02719
		impA/tssA	orf01365
		ompA	orf02730
		sciN/tssJ	orf02715
		tli1	orf02723; orf02724; orf02725
		tssF	orf02717
		tssG	orf02716
		vasE/tssK	orf02732
		vgrG/tssI	orf01356; orf01369; orf01373; orf02727
		vipA/tssB	orf01349; orf02734
		vipB/tssC	orf01350; orf02733
	T6SS-II	clpV	orf01168
		impH	orf01367
		impJ	orf01351
		sciN	orf01368
		vasA/impG	orf01366
	T6SS-III	-	orf01848
		-	orf01878
		dotU	orf01846
		icmF	orf01872
		impA	orf01877
		impF	orf01876
		impG	orf01873
		impH	orf01874
		impJ	orf01845
		ompA	orf01847
		sciN	orf01875
		vgrG	orf01849

Table S7 Antimicrobial resistance genes detected in *K. pneumoniae* CGMCC 31459 (CARD/RGI & ResFinder)

Gene	Drug Class	Resistance Mechanism	% Identity	% Coverage	Localization	Notes / Reference
blaSHV-41	β-lactams	Class A β-lactamase	100.0	100.0	Chromosome	Confirmed by ResFinder (AF535129)
fosA6	Fosfomycin	Antibiotic inactivation (glutathione transferase)	98.8	100.0	Chromosome	Consistent with intrinsic resistance in *K. pneumoniae* (KU254579)
oqxA	Fluoroquinolone, Chloramphenicol, Trimethoprim	RND efflux pump	99.3	100.0	Chromosome	Operon with oqxB, confirmed by ResFinder (EU370913)
oqxB	Fluoroquinolone, Chloramphenicol, Trimethoprim	RND efflux pump	99.1	100.0	Chromosome	Operon with oqxA, confirmed by ResFinder (EU370913)
baeR	Multiple drugs	Efflux pump regulator (RND system)	93.8	100.0	Chromosome	Intrinsic efflux regulation system in *K. pneumoniae*

Table S8 Prophage regions predicted in *K. pneumoniae* CGMCC 31459

Region	Region Length	Completeness	Score	Total Proteins	Region Position	Most Common Phage	GC %
1	5.7Kb	incomplete	50	8	1145581-1151288	PHAGE_Klebsi_ST437_OXA245phi4.1_NC_049448 (2)	49.09%
2	11.3Kb	incomplete	60	12	1159275-1170623	PHAGE_Escher_500465_1_NC_049342(12)	54.32%
3	75.8Kb	intact	150	58	2883205-2959021	PHAGE_Entero_mEp237_NC_019704(8)	52.30%
4	8Kb	incomplete	20	12	4730760-4738803	PHAGE_Escher_503458_NC_049341(2)	57.91%

Table S9 Plasmids 1 identified in *K. pneumoniae* CGMCC 31459

Sequence	Length （bp）	value	Overall identity	Gaps	Query aligned	Subject aligned
VFG001248 (fleQ) transcriptional regulator FleQ [Flagella] [Pseudomonas aeruginosa PAO1]	1473	1e-04	100.0% (26/26)	0	183445-183470	728-703
VFG002076 (clpV1) type VI secretion system AAA+ family ATPase [HSI-I] [Pseudomonas aeruginosa PAO1]	2709	6e-04	93.9% (31/33)	0	120881-120913	2100-2132
VFG002480 (tssH-5/clpV) Clp-type ATPase chaperone protein [T6SS-1] [Burkholderia pseudomallei K96243]	3039	4e-08	90.8% (89/98)	0	119677-119724, 120226-120275	680-727, 1223-1272
VFG014984 (algW) AlgW protein [Alginate regulation] [Pseudomonas aeruginosa PAO1]	1170	0.002	87.5% (42/48)	0	132316-132363	646-693

Table S10 General Characteristics of the Genome Sequences Used in This Study

Strain name in GenBank (accession no.)	Total size (Mb)	Completeness (%)	Contigs	GC Contents (%)	No. of genes	Contamination rate (%)
K. pneumoniae CGMCC 31459 （GCA_049200385.1）	5.15	99.99	5	57.55	4776	0.07
K. pneumoniae NTUH-K2044 (GCA_000009885.1)	5.20	100	2	57.36	5018	0.7
K. *pneumoniae* MGH 78578 (GCA_000016305.1)	5.27	100	6	57.14	5290	0.33
K. pneumoniae HS11286 (GCA_000240185.2)	5.28	99.65	7	57.12	5451	0.19
K. pneumoniae UCI 56 (GCA_000694815.1)	5.40	99.7	4	57.04	4975	0.33
K. *pneumoniae* ATCC 43816 （GCA_000742755.1）	5.32	100	1	57.36	4915	0.11
K. pneumoniae XH209 (GCA_001699105.2)	5.07	100	3	57.27	5054	0.4
K. pneumoniae GN-2 (GCA_001939885.2)	5.34	100	2	57.10	5225	0.95
K. pneumoniae QS17-0029 (GCA_003073235.1)	5.27	100	7	57.09	5269	0.78
K. Pneumoniae CR-HvKP1 (GCA_005853785.1)	5.34	99.4	6	56.92	5543	0.02
K. pneumoniae K28074 (GCA_025397975.1)	5.34	99.7	2	57.34	5062	0.22
K. pneumoniae K30821 (GCA_025725705.1)	5.35	99.7	6	56.84	5572	0.22
K. pneumoniae HVKP1 (GCA_030020765.1)	5.21	100	4	57.21	5122	0.55

Table S11 Monosaccharide Composition of EPS-KP

Monosaccharide Composition and Area Integration Percentage/%			
Man	GlcUA	Glc	Gal
16.286	5.418	49.481	28.813
